# Unsupervised morphological segmentation of tissue compartments in histopathological images

**DOI:** 10.1371/journal.pone.0188717

**Published:** 2017-11-30

**Authors:** Shereen Fouad, David Randell, Antony Galton, Hisham Mehanna, Gabriel Landini

**Affiliations:** 1 School of Dentistry, Institute of Clinical Sciences, University of Birmingham, Birmingham, United Kingdom; 2 Department of Computer Science, University of Exeter, Exeter, United Kingdom; 3 Institute of Head and Neck Studies and Education, Institute of Cancer and Genomic Sciences, University of Birmingham, Birmingham, United Kingdom; Worcester Polytechnic Institute, UNITED STATES

## Abstract

Algorithmic segmentation of histologically relevant regions of tissues in digitized histopathological images is a critical step towards computer-assisted diagnosis and analysis. For example, automatic identification of epithelial and stromal tissues in images is important for spatial localisation and guidance in the analysis and characterisation of tumour micro-environment. Current segmentation approaches are based on supervised methods, which require extensive training data from high quality, manually annotated images. This is often difficult and costly to obtain. This paper presents an alternative data-independent framework based on unsupervised segmentation of oropharyngeal cancer tissue micro-arrays (TMAs). An automated segmentation algorithm based on mathematical morphology is first applied to light microscopy images stained with haematoxylin and eosin. This partitions the image into multiple binary ‘virtual-cells’, each enclosing a potential ‘nucleus’ (dark basins in the haematoxylin absorbance image). Colour and morphology measurements obtained from these virtual-cells as well as their enclosed nuclei are input into an advanced unsupervised learning model for the identification of epithelium and stromal tissues. Here we exploit two Consensus Clustering (CC) algorithms for the unsupervised recognition of tissue compartments, that consider the consensual opinion of a group of individual clustering algorithms. Unlike most unsupervised segmentation analyses, which depend on a single clustering method, the CC learning models allow for more robust and stable detection of tissue regions. The proposed framework performance has been evaluated on fifty-five hand-annotated tissue images of oropharyngeal tissues. Qualitative and quantitative results of the proposed segmentation algorithm compare favourably with eight popular tissue segmentation strategies. Furthermore, the unsupervised results obtained here outperform those obtained with individual clustering algorithms.

## Introduction

The automatic segmentation of cells and tissues in digitized histopathological images is an essential stage in computer-assisted analysis. Over the years, various approaches have been proposed integrating computer vision and machine learning tools, e.g. [1, 2]. Ideally, a first step is to partition the image into distinct regions; followed by machine learning methods that automatically detect patterns within those regions conforming to given histological models in order to guide further analysis and enable enable ‘intelligent’ procedures that respond to image contents.

There has been a growing interest in developing automatic routines for segmentation and classification of tissue compartments in images of H&E stained histological sections obtained by light microscopy. Several studies emphasise that determining accurately the spatial distribution of such tissues is essential for extracting results of prognostic value, e.g. cancer growth and progression and tumour microenvironement [3]. Therefore analysis of the sample make-up (typically epithelium and stroma in the samples investigated here) will serve for identification of specific areas (e.g. epithelial cancerous tissues) as well as the tumour progression in digital images. However, the large variability and complexity of histological samples makes this a challenging task, especially for the H&E stained images which, in addition, exhibit an element of variability in staining uptake.

There is a rich body of literature on automated segmentation of tissue compartments, particularly those represented by epithelial and stromal tissues. Current approaches utilize supervised segmentation methods (e.g. [4–7]) where predictive models are built from labelled training data to predict the classes of novel unlabelled data. While such techniques have delivered promising results, they have a number of limitations. Firstly, those methods often require large amounts of hand-labelled region annotations (delineated histological components by experts) for training purposes. In practice, obtaining detailed annotations on digital histopathology images is challenging and time-consuming. Secondly, most approaches exploit traditional segmentation strategies (e.g. they use fixed-size square windows or are pixel-based) to partition the image into multiple binary subregions prior to applying the predictive models. Such low-level image segmentation approaches can yield poor quality results (e.g. over/under-segmented images) and this affects the classification accuracy. Thirdly, the supervised learning models are unable to perform well in on-line learning settings, where only one data pass is allowed, that is on the raw unlabelled data. On-line learning is often needed in a variety of data stream problems, including real-time decision making and resource-constrained learning [8]. These problems sometimes arise in histopathological imaging analysis, where an instant decision is required on novel unlabelled data. From the machine learning perspective, these problems are often amenable to the application of unsupervised learning models.

Cluster analysis is an unsupervised task that seeks to partition unlabelled data samples into homogeneous clusters based on assumed similarity measures. In histological imaging, clustering is particularly useful as an exploratory tool as it can provide information about the hidden image structures that may support anatomical and diagnostic models (e.g. [9–11]). Unsupervised recognition approaches are attractive because, unlike supervised methods, they do not require predefined image data annotations, making clustering powerful in scope and potentially useful in the current context of histological imaging.

The machine learning literature covers several different types of clustering techniques, each with its own strengths and weaknesses; often these different approaches, when applied to the same dataset, will give rise to varying segmentation results, depending on the algorithms used. Furthermore, some clustering algorithms have been shown to be sensitive to initialisation parameter changes so that again, they may lead different cluster results are obtained for the same data when those parameters are adjusted [12, 13]. Although re-sampling and cross validation techniques help in optimising model parameters and assessing the stability, it is hard to find the “best” algorithm for a given data set. To overcome this, recent approaches have suggested collecting results from multiple clustering algorithms in a repository known as the ‘cluster ensemble’, and combining the multiple results into one robust solution. This approach is known as consensus clustering (CC) [14–16], and lends itself to imaging applications (e.g. [17, 18]). However, to the best of our knowledge, its application to histopathological imaging, and particularly to the segmentation of tissue compartments, remains to be fully explored.

**Fig 1:**
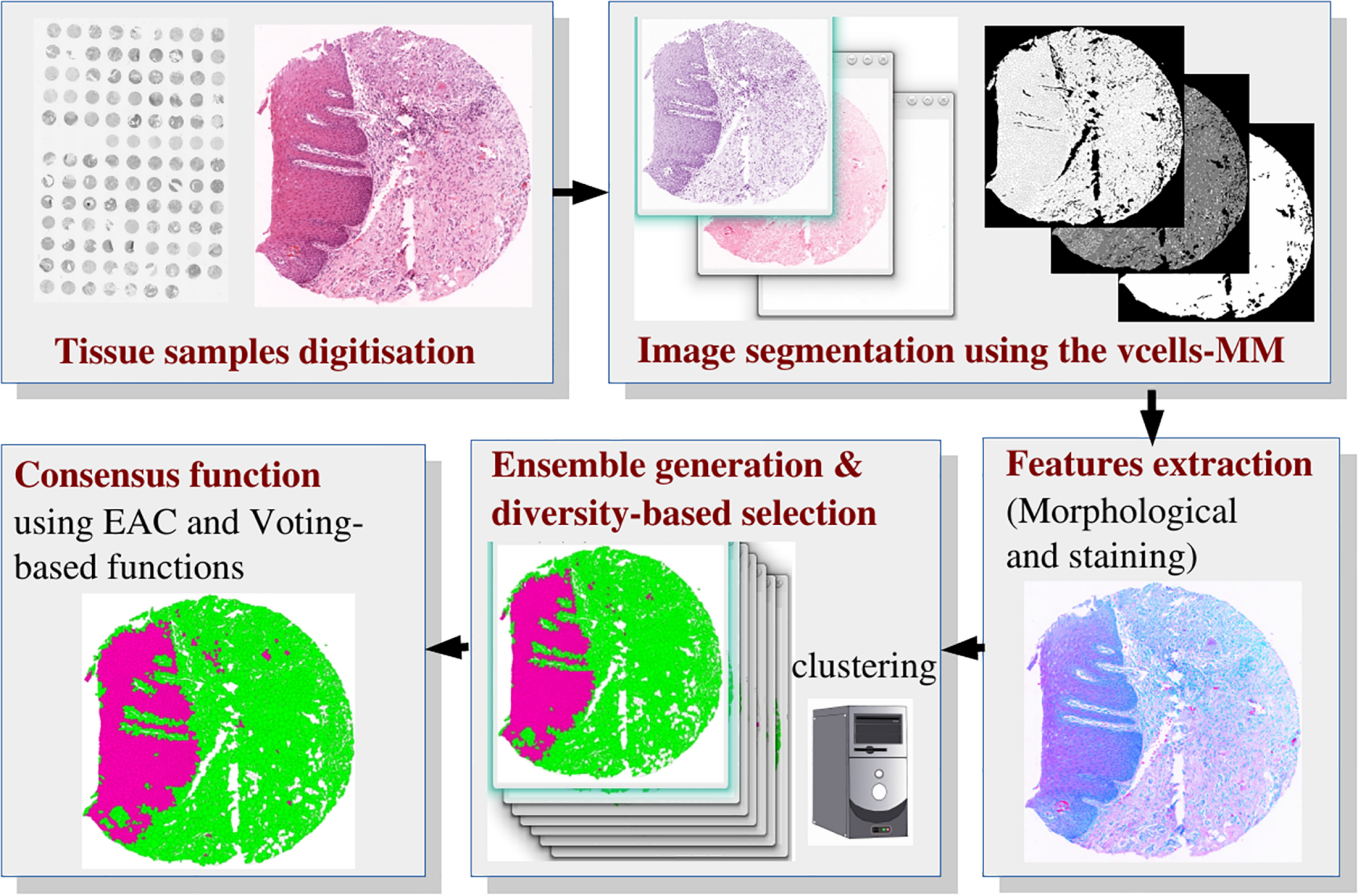
Block-diagram with an overview of the proposed method.

In this paper we present an unsupervised (data-independent) strategy for segmenting images into epithelial and stromal tissues, in haematoxylin and eosin (H&E) stained sections from tissue micro-array (TMA) cores. The first step is to partition the image into distinct regions; this is followed by the use of unsupervised learning models, based on the CC method, to automatically detect tissue compartments in images without the necessity for prior annotation of training data (hand annotated images) that is often required in supervised models. A block-diagram with an overview of the proposed method is presented in Fig 1. The contributions of the proposed framework can be summarized as follows:

1. We introduce a novel initial segmentation algorithm based on mathematical morphology [19], to partition images into binary regions or tiles representing potentially relevant image segments. Unlike most of the current segmentation methods, our approach reduces the image complexity while capturing some intended abstracted model of reality (i.e., virtual-cells, each enclosing a potential nucleus).
2. We utilize colour features which quantify the distribution of the dye uptake (haematoxylin, eosin and a residual channel) within those segmented regions, instead of exploiting the standard red, green, and blue components of the image. We also present a method that extracts and combines morphological features from the segmented virtual-cells as well as their enclosed nuclei.
3. We exploit a consensus clustering framework for the unsupervised recognition of epithelial and stromal regions to provide a robust unsupervised identification of different tissue compartments. This is in contrast to most of the existing unsupervised histological segmentation approaches that utilize a single clustering method, which affects the reliability and robustness of the results (e.g. [9–11]). Two consensus functions are considered here, the Evidence Accumulation Clustering (EAC) [14] and the voting-based consensus function (e.g. [16]). We also suggest an ensemble selection procedure for selecting an effective cluster ensemble based on diversity measures.
4. We propose a implementation for the voting-based consensus method, based on image processing operations, to generate a consistent labelling scheme among the base clustering outcomes. A preliminarily version of the voting-based algorithm applied to other segmentation methods, not covered in this paper, will be presented (with different findings) at a forthcoming conference [20].

This paper is organised as follows: Section 1 gives the background and briefly describes relevant previous work. Section 2 gives a brief description of the data used and preparation process. Sections 3, 4 and 5 describe our unsupervised segmentation, feature extraction and CC methods, respectively. Experimental results are presented in section 6 and discussed in section 7. Section 8 concludes the study.

## 1 Related work

The various approaches used in histological image segmentation depend on the nature of the analyses required and the features needing identification, e.g. nuclei, cells, higher hierarchical structures such as glands, tissue types where cells reside (compartments), the relations they hold and any histological or diagnostic types they might represent. The attributes of those histologically-relevant regions are often quantified on the basis of size, shape, colour intensity and texture. For this purpose, classical segmentation techniques are often based on pixel-based routines, e.g., intensity thresholding to identify differentially stained structures as foreground objects [21]. More advanced algorithms (such as the marker watershed, hidden markov models, grow-cut, seeded region growing and geodesic active contour) have been used to target more complex scenarios [22–25]. Algorithms have also been used to address the problem of partial occlusion and merged nuclei, e.g. [26]. Image segments resulting from these segmentation steps can be characterized using multi-dimensional feature sets, which in turn can be fed to supervised or unsupervised learning models in order to determine their nature, identify abnormalities or group them into different histological types or grades of disease. Comprehensive surveys of image segmentation techniques are presented in [1, 2].

The automated identification of stromal and epithelial regions within H&E tissue images has recently received increased attention in the histopathological analysis community owing to pervasive use of this type of material in diagnostic pathology. Current approaches often exploit traditional binary segmentation to select patches from large images prior to feature extraction. Then they apply supervised learning models which attempt to discriminate between the classes of interest by learning from a large training data set (predefined hand-annotated histological images). In [4], texture features (based on local binary patterns) and a SVM classifier have been exploited for the automated segmentation of epithelial and stromal tissues in TMAs of colorectal cancer. The initial segmentation was performed by downscaling the images by 50% and then partitioning it into square blocks. Binary classification of epithelial and stromal tissue was then performed by the SVM model which processes the blocks independently. Hiary et al. [5] proposed a Bayesian voting-based model for automated segmentation of stromal tissue from immunohistochemistry (IHC) images of breast cancer. The study utilized colour-based texture features extracted from predefined square image blocks. A feature learning based on deep convolutional neural networks was presented in [6] to automatically segment and classify epithelial from stromal tissues in H&E and IHC images of breast and colon cancer. Unlike other approaches that are based on hand-crafted feature representation, such as colour and texture, the DCNN used a deep architecture to learn high-level features in a data-driven fashion. The extracted features were used to construct a supervised classifier for discriminating the two types of tissues. However, deep learning models generally require large amount of training data and therefore it is computationally expensive to train. Wang et al. [7] present a pixel-based supervised segmentation method, using texture features, which segments H&E tissue images into four classes of tissue morphologies, including tumour, stroma, lymphoid/inflammatory cells/necrosis and background.

Several approaches in the context of unsupervised segmentation have used clustering analysis. In [9] a hierarchical self-organizing map clustering algorithm was exploited to segment H&E stained images of prostate tissues and identify four tissue clusters (glands, epithelium, stroma, and cell nuclei) based on colour and textural features. Chen et al. [10] used a multi-level fuzzy c-means clustering algorithm to identify cell nuclei, stroma and fat-like regions that enabled the detection of potential cancerous cells in virtual slide images. Naqvi and Garibaldi [11] studied unsupervised learning methods as objective replacements for breast cancer grading using principal component analysis for data dimensionality reduction, followed by fuzzy c-means clustering algorithm for automatic determination of cancer grades.

Owing to the high complexity of histological images, unsupervised segmentation using clustering methods remains a challenging task because performance often relies on single clustering algorithms and these tend to behave differently with various types of images. Here we suggest an improved unsupervised histological segmentation that aggregates the strengths of several individual clustering algorithms. In this so-called Consensus Clustering (CC) approach, the results of different clusterings are combined into a single, more robust and stable solution. A thorough summary of the existing CC approaches is presented in [15].

While CC has received attention in the machine learning community, it has not been efficiently exploited in the context of unsupervised histological imaging. Khan et al [17] proposed a framework for random projections of data features with ensemble clustering and applied it to pixel-level classification of tumour vs. non-tumour regions in breast cancer images. In this case, multiple random projections of features were used to generate multiple clustering results from the low dimensional representation of features and a consensus function combined the partitions and generated a final consensus result. However, the consensus utilised a single pixel-level clustering algorithm (the standard *k*-means [27]). Cooper et al. [18], used the CC model to aggregate tumours into groups based on their morphological signatures derived from the analysis of cell images.

## 2 Tissue samples, digitisation and preprocessing

Our analysis was performed on images of H&E stained tissue cores from oropharyngeal cancer TMAs prepared at the Institute of Cancer and Genomic Sciences, University of Birmingham, UK (the research was carried out under REC ethics reference 10/h1210/9, field Permit Institute for Head and Neck Studies and Education (InHANSE), University of Birmingham, UK). H&E is the routine staining method used in diagnostic microscopy; the haematoxylin dye is primarily taken up by nucleic acids (staining the nuclei blue/violet) and the eosin acts as counter-stain and stains in pink protein-rich material in the intra- and extra-cellular compartments. TMA slides were scanned using an Olympus BX50 microscope (Olympus Optical Co. Ltd, Tokyo, Japan) with a x20 magnification objective (N.A. 0.5, resolution 0.67 *μ*m), attached to a QImaging Retiga 2000R camera with a liquid crystal RGB filter (Surrey, BC, Canada). A motorised stage (OASIS Glide XY Scanning Stage, Objective Imaging, Cambridge, UK) and a Prior H122 (Prior Scientific, Cambridge, UK) motorised focus drive were controlled by the Surveyor (Object Imaging) platform which scanned the image and automatically stitched multiple fields. Before stitching, each field was background-corrected using the light transmittance as the ratio of transmitted light through the specimen and the incident light, scaled by the bit depth of the image (for each of the RGB components). The individual core images were approximately 3300 × 3300 pixels in size and the inter-pixel distance was 0.367*μ*m. We selected fifty-five images to be used in the analysis (ten images were used for the optimization of model parameters and forty-five images were used for testing). The selected images had substantial variation in their appearance as well as their tissue distribution (2.3% to 98.8% of epithelium tissue component and 25.5% to 83.2% of background out of the whole image).

The images were cropped to remove uninformative background areas around the tissue cores. The purpose of this procedure was to reduce the burden on the computational processing required for the subsequent tasks. This was achieved by using a thresholded version of the images, followed by a morphological opening by reconstruction, so that only the large portions of stained tissue are represented as foreground. Then the binary image was scanned from the boundary regions inwards until foreground pixels were found. Their coordinates were then used to define a new frame for the image and the image cropped.

## 3 Initial segmentation using mathematical morphology

**Fig 2:**
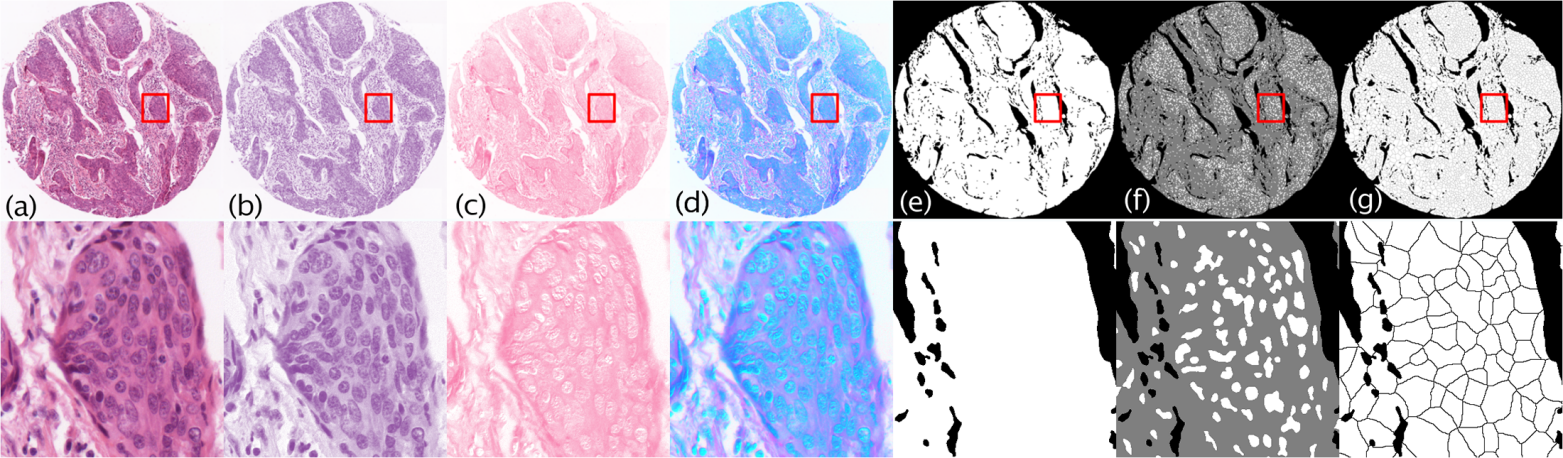
Initial segmentation steps using mathematical morphology. The top row illustrates the v-cells-MM segmentation on a TMA core (image width 3047 pixels, field width 1.19*mm*). The bottom row shows the details of a sub region (corresponding to the red region of interest in the top row). (a) H&E stained tissue, *I*. (b) Haematoxylin channel. (c) Eosin channel. (d) Stain RGB image, I*. (e) Foreground mask. (f) Nuclear markers (seeds) in white. (g) Segmented image *S* with v-cells-MM.

This section describes our segmentation based on Mathematical Morphology (MM) routines [19], that partitions an image *I* (Fig 2(a)) into a number of binary segments or ‘virtual-cells’ (v-cells) represented in image *S* (Fig 2(g)). The algorithm, denoted here as “vcells-MM”, can be described in four main steps as follows:

1. **Colour deconvolution.** This procedure separates the contribution of the two (H&E) dyes to exploit the differential stain uptake to identify the different structures in the image, in a similar way to how human observers interpret stained sections (e.g. nuclei are more strongly stained by haematoxylin than cystoplasmic and extracelluar stromal material, etc.). Each pixel in *I* has colour information represented as red, green, and blue channels as captured by the imaging equipment. Colour deconvolution [28] can process the RGB information from two or three combined dyes (in our case, haematoxylin and eosin) into separate ‘stain channels’ (see Fig 2(b) and 2(c)) if the original RGB components of separate dyes are known, assuming they combine subtractively (i.e. they are light absorbing dyes following the Beer-Lambert law [29]). In the case of two-dye stains, a third component represents a residual channel of the deconvolution process. The results of the deconvolution process can then be combined into a “stain” RGB image here denoted I* (see Fig 2(d)), where the R, G and B image channels hold the light transmittance of the haematoxylin, eosin and residual images, instead of containing their red, green, and blue components. Note that this image (I*) is used for the forthcoming feature extraction step.
2. **Generation of the tissue mask.** A set of operations is applied to I* to generate a mask representing the stained tissue regions (i.e. the foreground of the image, Fig 2(e)). This is given by the minimum of haematoxylin and eosin channels. Huang’s fuzzy global thresholding [30] is then applied to obtain a binary mask and exclude the background. Unlike in other segmentation techniques (e.g. watershed [31], superpixels [32, 33]), which segment the whole image (background and foreground) and result in a large number of regions being processed, our method ensures that the subsequent partitioning process is only applied on the image foreground, thereby reducing the number of segments to process and improving the performance of the procedure.
3. **Extraction of nuclear markers (seeds).** This is achieved by finding the regional minima (dark regions) in the haematoxylin image (Fig 2(b)) using greyscale reconstruction (see [34]) for extracting dark “basins” of a given depth *h* in the greyscale space. The procedure is also known as *h*-concave transform [19] and is obtained by subtracting the original image from the *h*-minima transform. These regions are dark 8-connected regions of up to the given depth *h*, surrounded by strictly higher greyscale value pixels. Note that *h* controls the amount of detail in the image. A large *h* (deep basins) results in detection of darkly stained structures (in this case cell nuclei) while small *h* results in excessive amount of detail detected, leading to over-segmentation. The value of *h* was tuned experimentally over the range of [10, 100] using a cross validation procedure in order to find an optimum value. The binary image containing the set of nuclear seeds is shown in Fig 2(f). Small regions attributable to noise (which would lead to over-segmentation) were removed using a binary opening by reconstruction. This consists of deleting regions that disappear after a number of morphological erosions *e* and it is achieved by eroding the mask image *e* times and reconstructing the original using the eroded image as seeds. This advantage of this over traditional morphological opening is that it deletes small regions without smoothing the remaining ones.
4. **Partitioning of the tissue mask into v-cells.** In this step, mask pixels are assigned to exclusive zones of influence for each seed; using the watershed transform [35, 36]. The segmented image *S* (see Fig 2(g)) is finally saved for further processing. Note that the size and the number of the generated v-cells is controlled by the value of *h*, as discussed above.

## 4 Feature extraction and selection

The purpose of this step is to extract a set of features from the segmented regions in *S* to perform clustering using a machine learning framework. As shown in previous works (e.g. [4–7, 10]), colour and morphological features are carriers of important structural information in histological images. While H&E is not a stoichiometric staining method, the differences in dye uptake across histological structures achieved with standardised laboratory protocols is sufficient to enable humans to recognise features unequivocally in relation to the differences in the composition of tissues, e.g. identifying different tissue compartments in a section. We investigated the distribution of dye uptake in the v-cells using eleven descriptors for each dye (haematoxylin, eosin and residual channels) from image I*. However, colour features alone were not sufficient to obtain high clustering accuracy. Therefore, we selected a set of morphological descriptors for the v-cells as well as the nuclear markers (seeds) located inside the v-cells, ensuring that the morphological features obtained from each v-cell is correctly combined with those from their enclosed nucleus. This was achieved by first labelling each v-cell with a unique value and then assigning the same label to the enclosed nucleus. Experimental observations showed that inclusion of morphological features alongside colour features resulted in superior clustering results than using colour features alone. For the feature extraction, we used an ImageJ plugin in [37] for estimating various statistics of binary 8-connected segmented regions. A brief description of the used features is provided in Table 1.

**Table 1:** Details of the features used in the analysis [37].

Feature Category	Features List
**Colour features of v-cells**, computed for each dye channel	*modal* intensity value in each region. *median* intensity value in each region. *average* intensity value in each region. *average deviation* of the intensity values in each region. *standard deviation* of the intensity values in each region. *minimum* intensity value in each region. *maximum* intensity value in each region. *variance* of the intensity values in each region. *skewness* of the intensity values in each region. *kurtosis* of the intensity values in each region. *entropy* of the intensity values in each region.
**Morphological features of v-cells**	*perimeter*, polygon computed from the boundary pixels of a region. *area* inside the polygon defined by the perimeter. *MinR*, radius of the inscribed circle centred at the centre of mass. *MaxR*, radius of the enclosing circle centred at the centre of mass. *feret*, largest axis length. *breadth*, the largest axis perpendicular to the feret. *convex hull* calculated from boundary pixels. *chullArea*, area of the convex hull polygon. *aspect ratio* = *feret*/*breadth*. *circularity* = 4 * *π* * *area*/*perimeter*^2^. *roundness* = 4 * *area*/(*π* * *feret*^2^). $area\;equivalent\;diameter=\sqrt{\left(4/\pi \right)*area}$. *perimeter equivalent diameter* = *perimeter*/*π*. *equivalent ellipse area* = (*π* * *feret* * *breadth*)/4. $compactness=\sqrt{\left(4/\pi \right)*area)}/feret$. *solidity* = *area*/(*convex area*). *shape* = *perimeter*^2^/*area*. *rfactor* = *convex hull*/(*feret* * π). *modification ratio* = (2 * *MinR*)/*feret*. *sphericity* = *MinR*/*MaxR*. *ArBBox* = *feret* * *breadth*, area of bounding box along feret diameter. *rectangularity* = *area*/*ArBBox*.
**Morphological features of nuclear markers**	*perimeter*, described above. *area*, described above. *chullArea*, described above. *circularity*, described above. *solidity*, described above. *concavity* = (*convex area*) − *area*. *convexity* = (*convex hull*)/*perimeter*. *shape*, described above.

There is a large number of possible features to be extracted from our images, so we used a feature selection algorithm applied to a validation set (ten H&E labelled images) in order to remove the redundant and non-discriminative features. In particular, we used the Weka ‘feature subset evaluator’ (CfsSubsetEval) [38], to compute the worth of a subset of features by considering their relevance with respect to the prediction process along with the degree of redundancy between the features. The optimal set consisted of sixty three features and these are presented in Table 1.

## 5 Consensus clustering (CC) framework

Consensus Clustering (CC) is an elaboration of the classical clustering problem that aims to address the variable performance of single clustering algorithms under the assumption that the consensual opinion of a group of solutions is more reliable than the opinion of just one. In this approach, results of various clustering solutions are combined into a single consensus partition without accessing the features or algorithms that were used to obtain the individual clusterings. This improves the robustness, stability and quality of the unsupervised segmentation. The CC framework consists of three steps: (a) *ensemble generation* receives the input dataset and returns an ensemble of cluster solutions; (b) in *ensemble selection*, an effective sub-set of cluster solutions is chosen from the ensemble based on their diversity measure, and finally (c) the *consensus function* combines the solutions into a single, more robust clustering result.

### 5.1 Ensemble generation

Let *X* = {*x*_1_, *x*_2_, …, *x*_*n*_} be a set of *n* v-cells in the binary segmented image *S*. Note that each *x*_*i*_ is defined over the 63 features described earlier. In this study, a clustering algorithm takes *X* as an input and groups the *n* segments into (at least) two clusters corresponding to stroma and epithelium regions, forming a data partition *L*.

In this step, a number *C* of partitions (clustering results) are created for the same dataset *X*, forming the cluster ensemble *E*, where *E* = {*L*_1_, *L*_2_, … *L*_*C*_}, *L*_*i*_ = {*c*_*ji*_}, and *c*_*ji*_ is the *j*th cluster in *i*th data partition (*L*_*i*_). Partitions are obtained from different clustering algorithms where each runs multiple times while varying their parameters. In this study we used five commonly used clustering algorithms that exploit different clustering strategies to ensure diversity in the ensemble. These were: (1) ***k*-means** [27], a centroid based algorithm, (2) **Unsupervised Learning Vector Quantization (LVQ)** [39], a LVQ algorithm for unsupervised learning, (3) **Expectation Maximization (EM)** [40], a distribution based method (4) **Make Density Based (MDB)** [41], a density based algorithm, and (5) **Agglomerative Hierarchical Clustering (AH)** [42], a pairwise distance based approach.

### 5.2 Ensemble selection

The level of diversity between the ensemble members has been identified as an important factor in the performance of consensus clustering [43, 44]. Sometimes the generated ensemble includes a set of identical or irrelevant partitions. An ensemble of duplicate partitions will not outperform the individual ensemble members and hence there would be no need to apply a CC method. Conversely, an ensemble incorporating a few significantly inconsistent partitions (poorly correlated with respect to the rest of the ensemble) may lead to unreliable consensus output. The problem of generating a better performing cluster ensemble, using diversity-related heuristics, has recently received attention in the scientific community (e.g. [43]). In [44] it was shown that better consensus results are obtained when ensembles have moderate diversity among their partitions. Here we explored the dissimilarity among ensemble members to obtain an effective cluster ensemble, denoted $\dot{E}$, from the original pool of partitions *E*. The procedure aims at maintaining a moderate level of diversity among the partitions by pruning out significantly inconsistent partitions as well as identical or low diversity partitions and it is as follows:

Given clustering solutions *L*_*i*_ in the original ensemble *E*, to decide whether to include *L*_*i*_ in $\dot{E}$ we measure how well *L*_*i*_ agrees with the general trend contained in the original ensemble. The diversity of a given partition *L*_*i*_ is calculated using the average Rand Index (RI) [45] between *L*_*i*_ and each clustering solution *L*_*j*_ contained in *E*, where *i* = 1, ⋯ *C*, as follows:

$$\begin{array}{c} \text{similarity}\left(L_{i},E\right)=\frac{1}{C-1}\sum_{j=1}^{C}RI\left(L_{i},L_{j}\right), \end{array}$$ (1)

where (*L*_*i*_, *L*_*j*_ ∈ *E*) and (*i* ≠ *j*). The RI counts pairs of points where two clusterings agree or disagree and it is calculated as:

$$\begin{array}{c} \text{RI}\left(L_{i},L_{j}\right)=\frac{TP+TN}{TP+FP+TN+FN}, \end{array}$$ (2)

where *TP* and *TN* are the number of point pairs that are grouped in the same cluster, and in different clusters, respectively, under both *L*_*i*_ and *L*_*j*_. *FP* and *FN* are the number of point pairs that are grouped in the same cluster under *L*_*i*_ but not under *L*_*j*_, and in same cluster under *L*_*j*_ but not under *L*_*i*_, respectively. The RI lies between 0 and 1, where 1 implies two partitions agreeing perfectly, and 0 that they completely disagree.

Two thresholds *D*_1_ and *D*_2_ are defined corresponding to the minimum and maximum accepted level of diversity among the partitions. If *L*_*i*_ exhibits an acceptable level of diversity with respect to the rest of the population in *E* (i.e., larger than *D*_1_ and less than *D*_2_) then it is considered as an eligible voter and is added to the new ensemble, if the opposite applies then it is taken out from the new ensemble, as shown below, and replaced with another solution that satisfies the criteria. Note that it is not always possible to find another partition of the same algorithm that satisfies the criterion. In this case, the partition is removed without replacement. The total number of selected partitions in $\dot{E}$ is denoted here as $\dot{C}$, where $\dot{C}\leq C$, $\dot{E}$ is formed as follows,

$$\begin{array}{c} \dot{E}=\left\{L_{i}\;|\;\text{similarity}\left(L_{i},E\right)\in \left[D_{1},D_{2}\right]\right\} \end{array}$$ (3)

It is worth mentioning that we tried applying the CC methods (to be discussed below) on the original ensemble *E* by skipping the ensemble selection step, however, this yielded lower clustering accuracy when compared to the case of exploiting the new ensemble $\dot{E}$.

### 5.3 Consensus function

The goal of the consensus step is to find an *optimal* data partition, denoted here as L*, which represents the CC based on the information contained in $\dot{E}$. For this we use two consensus functions described below.

#### 5.3.1 Evidence Accumulation Consensus (EAC) function

The EAC algorithm [14] uses a voting mechanism that considers the co-occurrences of pairs of patterns in the same cluster as votes for their association. Note that the estimation of voting between partitions is deemed non-trivial owing to the usage of arbitrary cluster labels. To this end, the EAC uses a pairwise inspection approach to construct a new similarity measure between patterns through an *n* × *n* consensus matrix, defined as $M_{ij}=m_{ij}/\dot{C}$, where *m*_*ij*_ is the number of times the pattern pair (*i*, *j*) are grouped together in the same cluster and $\dot{C}$ is the number of cluster solutions in ensemble $\dot{E}$. Note that the consensus matrix *M* is a symmetric matrix of real numbers within the range from 1 (perfect consensus among the partitions) down to 0 (no association). The entries of *M* are treated as similarities and in order to produce L* we run another clustering algorithm on *M*. Here the Agglomerative Hierarchical Clustering (AH) algorithm was applied to extract L* from *M*. Ideally, L* should be consistent with the clustering results in ensemble $\dot{E}$ and exhibit robustness to small variations in results. The clustering output is represented in another image, namely $\dot{S}$.

#### 5.3.2 Voting-based consensus function

Unlike in classification tasks, labels resulting from clustering methods are symbolic, and therefore an individual partition (clustering solution) in $\dot{E}$ includes clusters that do not necessarily have labels that correspond to other clusters in different partitions of $\dot{E}$. The voting-based consensus function initially addresses the cluster label mismatch problem, and uses a simple majority voting technique to find the L* that optimally summarizes $\dot{E}$. The label mismatch is defined as the problem of finding the optimal re-labelling of a given partition *L*_*i*_ with respect to a reference partition *L*_*j*_. This problem is commonly formulated as a weighted bipartite matching problem [16], and it is solved by inspecting whether data patterns in two partitions share labels more than with other clusters.

In this paper we propose a re-labelling algorithm (based on image processing tools) to solve the labelling mismatch problem among the partitions in $\dot{E}$. Recall that data points in each partition represent image regions in the binary segmented image *S*. Labels resulting from the different partitions in $\dot{E}$ are assigned to the corresponding image regions in *S* using two different colours. The labelled regions are displayed in two images denoted here as $IMG_{L_{i}}$ and $IMG_{L_{j}}$ for partition *L*_*i*_ and *L*_*j*_, respectively. As a result of label mismatching, however, a pair of correlated clusters from different partitions may be assigned different labels in the images. Our target is therefore to permute the cluster labels so as to maximise agreement amongst the labels within each partition of $\dot{E}$. Specifically, epithelial tissue regions should have the same label across all partitions in $\dot{E}$, and hence appear in similar colours in their corresponding image regions, and the same applies to the stromal regions.

The algorithm basically exploits the fact that pairs of individual clusters from different partitions, *L*_*i*_ and *L*_*j*_ match when the majority of their pixels overlap, in $IMG_{L_{i}}$ and $IMG_{L_{j}}$, respectively. To this end, individual clusters, denoted here as *c* and *c*′, found in partitions *L*_*i*_ and *L*_*j*_ are visualized in two separate binary images, denoted here as $IMG_{L_{i}\;c}$ and $IMG_{L_{j}\;c\prime }$, respectively. The algorithm then estimates the degree of overlapping/similarity between the two binary images, using the Jaccard Index (JI) evaluation measure [45] as follows:

$$\begin{array}{c} {\text{JI}}_{IMG_{L_{i}c},IMG_{L_{j}c^{\prime }}}=\frac{|IMG_{L_{i}c}\cap IMG_{L_{j}c^{\prime }}|}{|IMG_{L_{i}c}\cup IMG_{L_{j}c^{\prime }}|}, \end{array}$$ (4)

The JI value lies within the range of [0, 1], where 1 means a perfect correspondence between the two images and 0 implies zero correspondence.

For every cluster *c* in *L*_*i*_ we compute JI obtained against the other clusters in *L*_*j*_. Then, we inspect the cluster of the highest JI, if it has a different label (*c* ≠ *c*′) then the match is achieved by swapping the labels in the image $IMG_{L_{j}}$ and therefore the labels in *L*_*j*_. The process is repeated until the labels in the ensemble partitions are matched with the labels in the reference partition *L*_*i*_. Note that *L*_*i*_ remains unchanged throughout the re-labelling process. The now aligned labels for all partitions are combined into a final consensus partition L* via a majority voting technique. In exceptional cases, where the numbers of votes are equal we select the vote of the partitions that produce the highest total similarity (RI) with respect to the ensemble (Eq (2)). As before, L* will be represented in image $\dot{S}$.

Note that the suggested Voting-based significantly outperforms the existing EAC in terms of execution time. This is because, while EAC and Voting-based have complexity functions of order *O*(*n*^2^) and *O*(*K*^2^), respectively (where *K* is the number of classes), in our case *n* reached as high as 4000 in some images, but in every case *K* = 2.

## 6 Experiments and results

The purpose of the following experiments is two-fold. First, using the same initial segmentation technique (vcells-MM) we illustrate how the CC algorithms (EAC, Voting-based) improve the accuracy of clustering, compared to individual clustering approaches. Second, we evaluate the impact of using our proposed vcells-MM methodology on the performance obtained against other popular segmentation approaches. In this paper, all imaging procedures and machine learning algorithms were implemented on the ImageJ platform [46] using the WEKA data mining JAVA libraries [47]. We used a computer with an Intel R core(TM) i7-4790 CPU running at 3.60GHZ, with 32GB of RAM and 64-bit Linux operating system.

### 6.1 Clustering evaluation methods

The quantitative evaluation of clustering results is problematic owing to the label correspondence problem. Therefore, the analysis does not necessarily provide a clear interpretation/labelling of (in our case) tissue types. Because of this unavoidable limitation and for reasons of qualitative evaluation, a total of fifty-five images were manually labelled by one of us (GL), with a background in Oral Pathology, into epithelium, connective stroma and background areas to serve as a gold-standard (denoted here as *R*).

Five external cluster measures were used which quantify the similarity between our clustering result $\dot{S}$ and the gold-standard *R*. However, the difference in nature and number of data patterns in *R* (pixels) and in $\dot{S}$ (v-cell regions) does not allow a direct quantitative comparison between them. To overcome this problem, we generated a further set of gold-standard images (here denoted *G*) from *R* by assigning to each of the generated v-cells in the binary image *S* the corresponding tissue type found in *R*. This procedure unifies the number of the compared units (v-cells) among the images (*G* and $\dot{S}$) and facilitates the quantitative comparisons. However, on a few occasions a single v-cell in *S* incorporates more than one tissue type from the corresponding image *R*, and in such cases we selected the most prevalent type as the label. The evaluation measures used here are as follows:

- **The Rand Index (RI)**: This is defined in Eq 1 and it compares the final clustering solution given in our segmented image $\dot{S}$ with a known reference partition (gold-standard *G*), that is generally based on the class labels associated with the data.
- **Precision**: It denotes the number of true positive results divided by the number of all positive results as follows:
  $$\begin{array}{c} \text{Precision}=\frac{TP}{TP+FP} \end{array}$$ (5)
- **Recall**: It is the number of true positive results divided by the number of positive results that should have been returned as follows:
  $$\begin{array}{c} \text{Recall}=\frac{TP}{TP+FN} \end{array}$$ (6)
  Precision estimates the quality, whereas recall estimates the quantity. In particular, high precision denotes more relevant results returned by the algorithm than irrelevant ones, whereas high recall denotes that the algorithm returned most of the relevant results [48].
- **F1-score**: It is a weighted average of the precision and recall and it is defined as:
  $$\begin{array}{c} \text{F}1-\text{score}=2\cdot \frac{\text{Precision}\cdot \text{Recall}}{\text{Precision}+\text{Recall}}\;, \end{array}$$ (7)
  F1-score is usually more significant than the RI, especially if you have an uneven class distribution. It reaches its best value at 1 and worst at 0.
- **Jaccard Index (JI)**: This is defined in Eq 4 and it is estimated here by dividing the number of pixels in the intersection of two images ($\dot{S}$ and *G*) by the number of pixels in their union.

### 6.2 Comparison with individual clustering methods

This section compares the unsupervised CC frameworks (EAC and Voting-based) against five standard clustering algorithms (previously used in the ensemble generation step) namely, *k*-means, LVQ, EM, MDB and AH.

Firstly, for each scanned image *I* we applied the vcells-MM algorithm which partitioned *I* into *n* v-cells and produced the binary segmented image *S*. According to a cross-validation procedure performed on a validation set of ten images, we set the two segmentation parameters *h* and *e* to 30 and 3, respectively. As mentioned before, the parameter *h* controls the number of generated v-cells. Secondly, we extracted a set of colour and morphological features described in section 4 from each v-cell in *S* as well as their enclosed nuclear markers. The extracted features were passed to the five clustering algorithms (mentioned above). We fixed the number of clusters to two in all experiments. The *k*-means and the EM algorithms were run 10 times with different initialisation parameters. The LVQ, MDB and AH were run 4, 1 and 6 times, respectively. The number of seeds in *k*-means and EM were randomly chosen from the range [10, 200]. The AH algorithm was used with two link types (Complete and Mean) and three different distance functions (Euclidean, Manhattan and Minkowski). We also tried other link types (single, centroid and average methods), but they returned poor clustering results. Learning rates in the LVQ algorithm were set at the values of 0.05, 0.07, 0.09 and 0.1. The ensemble generation process yielded a total of 31 clustering solutions, stored in the initial pool of cluster solutions, *E*. Then, we applied the diversity selection strategy to form another better performing ensemble $\dot{E}$, which pruned the significantly inconsistent partitions and also reduced the number of identical clustering solutions. The *D*1 and *D*2 acceptance thresholds were set to 0.5 and 0.9, respectively. Finally, we applied the two discussed consensus functions (EAC and Voting-based) which produced one consensual robust clustering solution L*.

**Fig 3:**
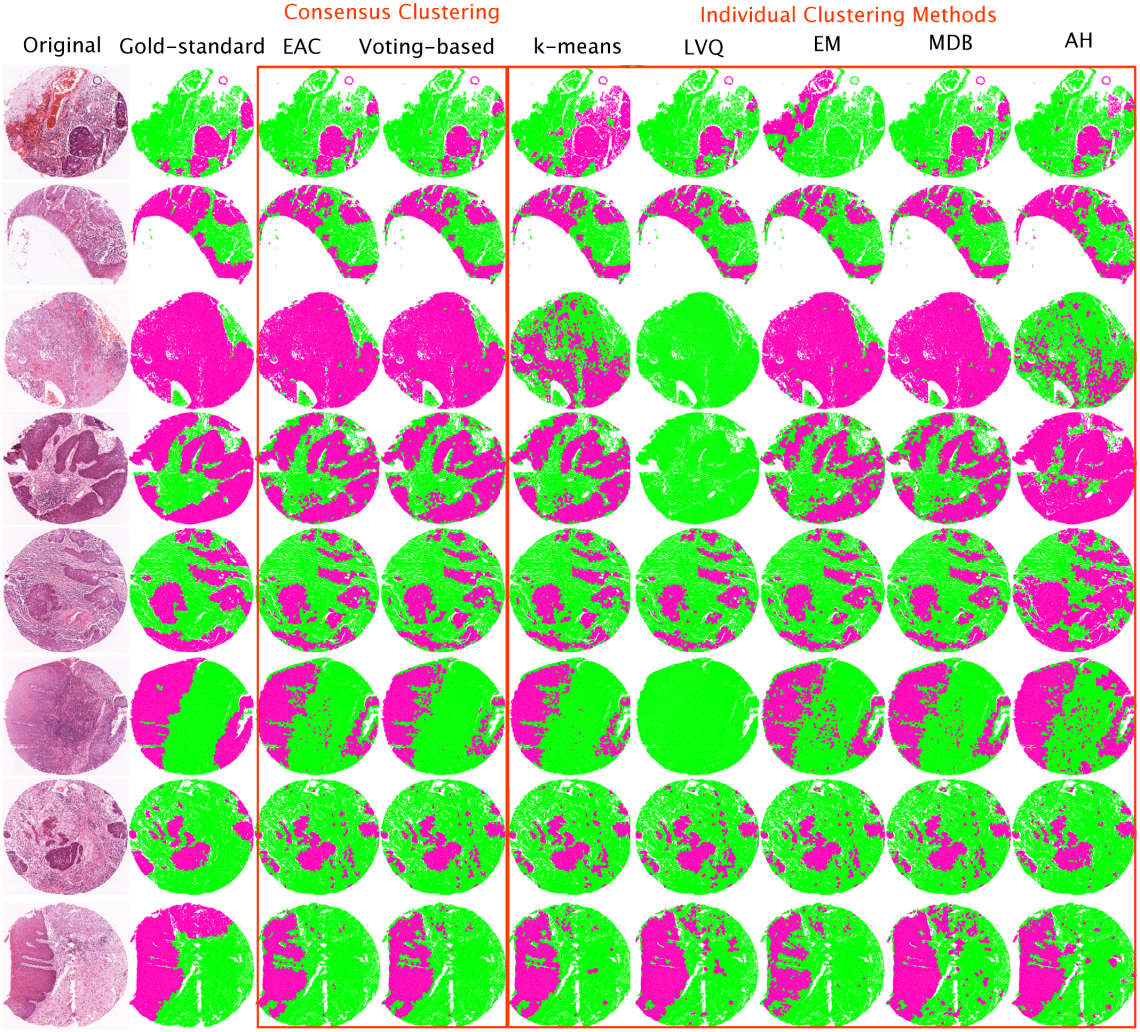
Evaluation output for the detection of regions in eight example H&E stained core tissue images (represented on the left of each row next to its corresponding gold-standard *G*). In all images, the vcells-MM framework is used in advance to produce the binary segmented images *S*. Each row displays seven different clustering results stated on the top of each image. In all images, the black, white, magenta and green colours correspond to the segmentation lines, background, epithelial regions, and connective stroma, respectively.

Quantitative and qualitative results of the EAC and Voting-based methods as well as the five individual clustering approaches are presented in Table 2 and Fig 3, respectively. To evaluate the performance of the individual clustering algorithms (Table 2), we evaluated the mean evaluation measures as well as the standard deviations across the forty-five test images for each algorithm run, then we estimated the average results across the multiple runs. Performance of the CC methods was evaluated by calculating the mean evaluation measures and the standard deviations across the forty-five test images. S1 Table provides a detailed version of the quantitative results for all the applied algorithms.

**Table 2:** Performance evaluation of the consensus clustering methods (EAC and Voting-based) compared against five individual clustering approaches in terms of mean RI, Precision, Recall, F1-score and JI along with standard deviations (±) across the forty-five tested images. In all algorithms initial segmentations are performed via our vcells-MM method. Best RI, F1-score and JI results are marked in bold font.

Measure	EAC	Voting-based	*k*-means	LVQ	EM	MDB	AH
RI (±)	**0.77** ± **(0.08)**	**0.77** ± **(0.07)**	0.74 ± (0.08)	0.65 ± (0.12)	0.73 ± (0.08)	0.74 ± (0.08)	0.73 ± (0.09)
Precision (±)	0.77 ± (0.07)	0.78 ± (0.07)	0.74 ± (0.08)	0.91 ± (0.11)	0.73 ± (0.09)	0.73 ± (0.07)	0.78 ± (0.11)
Recall (±)	0.84 ± (0.07)	0.84 ± (0.07)	0.82 ± (0.10)	0.66 ± (0.31)	0.82 ± (0.09)	0.84 ± (0.08)	0.77 ± (0.14)
F1-score (±)	0.80 ± (0.06)	**0.81** ± **(0.07)**	0.77 ± (0.08)	0.76 ± (0.09)	0.77 ± (0.07)	0.78 ± (0.07)	0.76 ± (0.09)
JI (±)	0.73 ± (0.08)	**0.74** ± **(0.08)**	0.67 ± (0.12)	0.40 ± (0.21)	0.67 ± (0.12)	0.70 ± (0.09)	0.63 ± (0.13)

For display purposes, in Fig 3 we randomly selected a single clustering output (out of the multiple runs) to represent the performance of the compared clustering algorithms. S1, S2, S3 and S4 Figs provide a detailed qualitative results for four example H&E stained tissue images. Each figure illustrates the initial segmentation steps used to partition a single tissue image into binary tiles. This is followed by showing all the clustering results obtained by the applied algorithms. We also provide in S6, S7, S8, S9, S10, S11, S12, S13 Figs and S2 Table a minimal anonymized data set with the resulting images in full resolution, as used in the study. The source code of our proposed methods is provided as a supporting file in S1 Source-code.

The overall result demonstrates the superiority of the EAC and Voting-based frameworks over the individual clustering approaches in detecting epithelial and stromal tissue regions. In particular, the highest accuracy was achieved by the EAC and Voting-based approaches (80% and 81% in terms of F1 score (respectively) and 77% in terms of RI for both). In terms of the comparison between EAC and Voting-based algorithms, Table 2 shows that the performance of Voting-based is slightly better than EAC, but more importantly (based on experimental observations) it is significantly faster due to its lower computational complexity. S5 Fig compares the time performance of the EAC against the Voting-based method across the forty-five tested images. The average time required by the EAC and the Voting-based to estimate the consensus clustering L* was 85.88 and 9.99 milliseconds, respectively.

Besides the discriminative ability, the visual comparisons provided in Fig 3 show that the EAC and Voting-based algorithms exhibit more consistent performance over individual clustering methods, as illustrated by lower standard deviations of the different evaluation measures. In particular, although most of the individual clusterings provide acceptable output across the displayed images, they all failed to perform well in at least one case. By contrast, the CC methods which show more stable clustering results.

### 6.3 Comparison with other segmentation methods

This section compares results of the colour-based clustering performance using the vcells-MM segmentation with those from other eight commonly used segmentation methods. The compared methods utilize various segmentation approaches and they can be categorised into three groups: **region-based**, **pixel-based** and **histogram-based segmentation**.

In **region-based segmentation**, regions are constructed by grouping homogeneous neighbouring pixels into regions. We assessed the performance of our method against the *watershed* [31], *waterfall* [49] and *superpixels* [32, 33].

The *watershed* transform takes its inspiration from geography. Given an irregular landscape flooded by water, the watersheds of a landscape define lines (dams) which divide the catchment areas (basins) that are filled with water. There are algorithms that implement this idea in the digital domain (e.g. [50]), although the classical watershed often leads to over-segmentation.

The *waterfall* algorithm is a hierarchical segmentation method [49] that aims to reduce the over-segmentation of the watershed transform. An initial watershed segmentation is processed iteratively to achieve increasingly simplified partitions by removing watershed dams between basins that are surrounded by higher basins. Thus, the importance of a basin dam line is considered with respect to its neighbouring basins. The process may be repeated until a final single region is obtained. The initial watershed segmentation was applied to the greyscale gradients of the original image. Then two consecutive levels (iterations) of the waterfall algorithm were applied, denoted as levels L1 and L2. Subsequent levels were also tried, but they returned grossly under-segmented results deemed not suitable for our purposes.

In *superpixels* segmentation, image pixels with similar colour and spatial properties are also grouped into atomic regions, so-called superpixels, which reduces the complexity of pixel grid images. Here we used an advanced version of the superpixels segmentation, namely Simple Linear Iterative Clustering (SLIC) framework [32, 33] which partitions the original image *I* into a set of compact and relatively uniform superpixels. The method allows the size and compactness of the resulting superpixels to be adjusted, providing some control over the number of superpixels generated. In our experiments, we used the size that provided the best clustering results on the validation set. We used the recently proposed jSLIC [51], a Java implementation of SLIC that is faster than the original version in [32].

**Fig 4:**
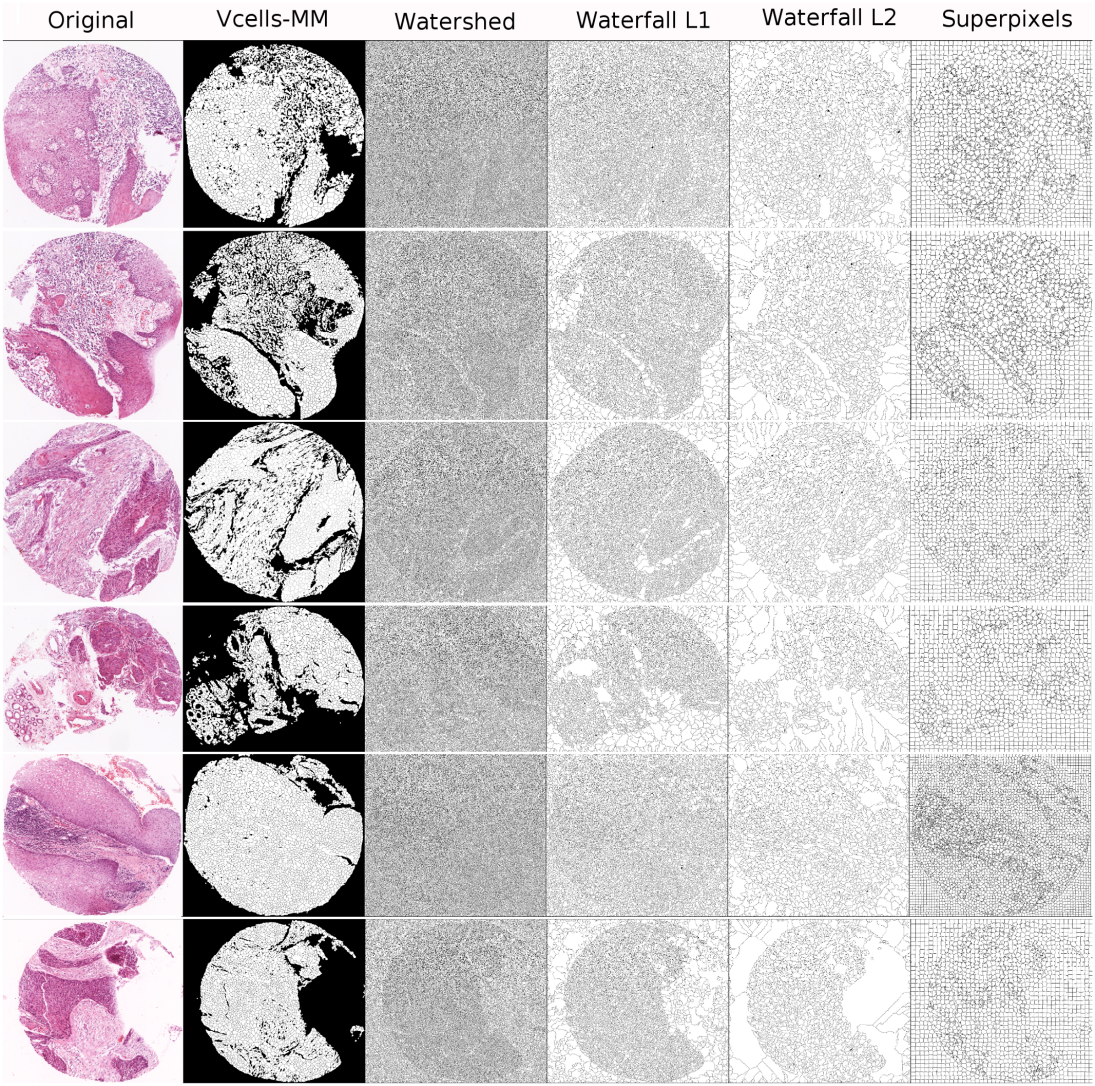
Evaluation output for the initial binary segmentations obtained by the region-based segmentation methods in six example H&E stained core tissue images. The first column (from the left) displays the original scanned image, the second presents the binary segmented images resulting from our vcells-MM method and the remaining columns displays the results obtained by the compared region-based segmentation approaches (each labelled above). In all images, the black colour corresponds to the segmentation lines.

In the three aforementioned methods, initial segments were firstly extracted from image *I* to generate a binary segmented image (see Fig 4). Colour features of the segmented regions were extracted and passed to the subsequent unsupervised recognition procedures. Note that, unlike the earlier experiment, we excluded morphological features because many of the resulting segments obtained with the other various methods did not represent comparable histological components across epithelial and stromal compartments (e.g. superpixels and pixel-based). Therefore, some binary segments can exhibit marked heterogeneity, which can affect the subsequent analysis. For fair comparisons among the segmentation approaches, clusterings were performed using just the *k*-means algorithm and not the CC methods, because in this comparison we wanted to highlight the advantages of the initial vcells-MM segmentation method alone. The number of randomized seeds used in *k*-means was fixed at 50 in all experiments.

The **pixel-based segmentation** method is based on a *k-means pixel clustering* using colour features (values from the RGB channels). Each pixel in the output image represents the cluster to which the original pixel was assigned. For this, we used the ImageJ pixel-based *k*-means clustering plugin [52].

**Histogram-based segmentation** methods are among the simplest and the most commonly used techniques in image segmentation. They operate on pixels based on the image intensity histogram. We tested the performance of three histogram-based segmentation methods and compared their segmentation results with the ones obtained by our approach. In particular, we tested the performance of the *multi Otsu thresholding* technique [53] (an extended version of the Otsu’s method [54]), which maximises the between-class variance of pixel intensity to partition the image. The extended multi Otsu thresholding generates multiple thresholds from an image. Note that our images contain three classes of (background, epithelial tissue and stromal tissues). We used the ImageJ multi Otsu Thresholding plugin available in [55] to segment the grey level images into three classes.

We also compared our results against a fuzzy-based thresholding approach known as ‘*no-threshold segmentation*’ [56] where grades of membership to *C* classes are estimated using a global probability density function, and the mode of the regularised histogram corresponds to one class of pixel. Here, we used three classes corresponding to our image regions of interest. This procedure produces three images, each presenting the grades of membership of every pixel to the three classes. The final segmentation is obtained by applying probabilistic relaxation to the images and defuzzification to the grades of membership. An ImageJ plugin for this purpose is available in [57].

Finally, the *maximum entropy multi-thresholding* technique had also been assessed against our segmentation method. It is a generalization of the single maximum entropy method that is presented in [58].

Note that owing to the complexity of our images, the *waterfall* segmentations required extremely long computation times. To improve this, we downscaled the original image *I* by 50% before applying all the above eight segmentation methods. For all segmentation methods, comparisons were done in terms of Jaccard Index obtained against the rescaled (by 50%) gold-standard image *R*. The JI was averaged across the forty-five test images for each segmentation method. The region-based methods are known for being computationally expensive, for this we compared the total execution time (in seconds) and the total number of segments generated for the region-based as well as the pixel-based methods.

Quantitative results (presented in Table 3) show that the unsupervised segmentation performance obtained by our vcells-MM framework outperforms the other segmentation methods. In particular, for the region-based segmentation methods and with respect to the mean JI, the vcells-MM approach achieved an average relative performance improvement of 19%, 28%, 25% and 13% over the *watershed*, *waterfall L1*, *waterfall L2* and *superpixels*, respectively. It also maintained the lowest mean number of segments as well as a reasonable execution time. For the pixel-based segmentation, our method achieved an average relative performance improvement of 28% over the *k-means pixel clustering*. For the histogram-based methods our algorithm attained a performance improvement of 25%, 23%, 76% over the *multi Otsu*, *no-threshold* and *maximum entropy multi-thresholding*, respectively.

**Table 3:** Performance evaluation of our vcells-MM segmentation compared against eight existing commonly used segmentation methods. The compared methods utilize various approaches including region-based segmentation, pixel-based segmentation and histogram-based segmentation. For all methods performance are evaluated in terms of mean Jaccard Index (JI) along with standard deviations (±) across forty-five tissue images. The table also reports the mean number of segments generated, as well as the mean execution time (in seconds) for region-based and pixel-based segmentations. Best JI result (the vcells-MM) is marked with bold font.

Segmentation approach	Method name	Number of segments	Execution time (sec)	JI (±)
**Region-based segmentation**	Vcells-MM	1679 v-cells	12.7 sec	**0.69** ± **(0.08)**
	watershed	58075 segments	70.54 sec	0.58 ± (0.09)
	waterfall L1	7585 segments	199.02 sec	0.54 ± (0.10)
	waterfall L2	1722 segments	293.44 sec	0.55 ± (0.13)
	superpixels	2817 Superpixels	2.6 sec	0.61 ± (0.12)
**Pixel-based segmentation**	*k*-means pixel clustering	4926428 pixels	6.80 sec	0.54 ± (0.07)
**Histogram-based segmentation**	multi Otsu thresholding	N/A	N/A	0.55 ± (0.13)
	no-threshold segmentation	N/A	N/A	0.56 ± (0.07)
	maximum entropy multi-thresholding	N/A	N/A	0.39 ± (0.07)

**Fig 5:**
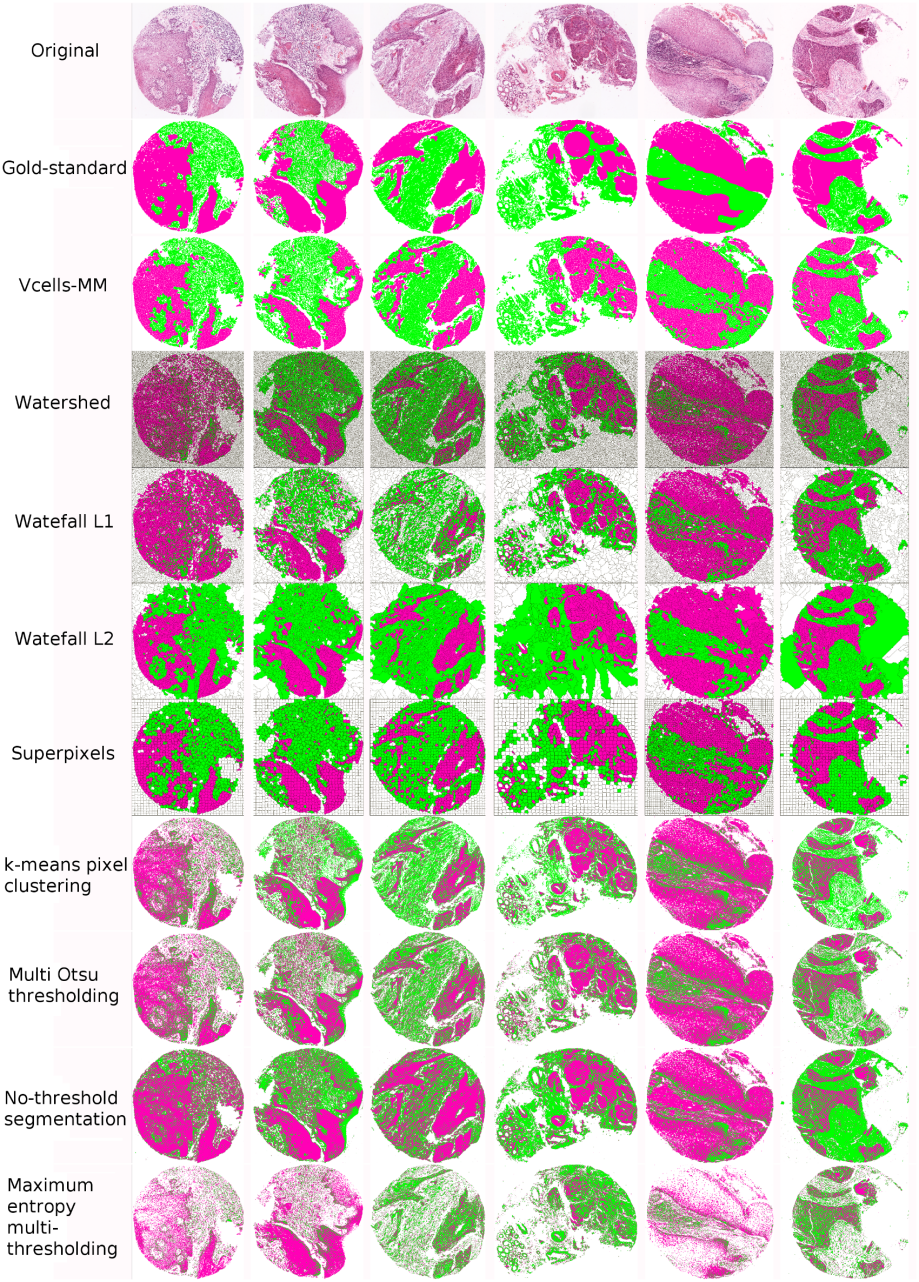
Evaluation output for the detection of epithelial and stromal regions in six example H&E stained core tissue images. The first row (from the top) displays the original scanned image, the second presents the gold-standard images *R*, the third shows our Vcells-MM segmentation results and the remaining rows depict the segmentation results obtained by the various methods (each labelled on the left). In all images, the black, magenta and green colours correspond to the segmentation lines, epithelium and stroma regions, respectively.

Qualitative results are presented in Figs 4 and 5. Fig 4 shows the binary segmented images of six example cases of H&E core tissue images. The segmented images are resulting from our vcells-MM method (second column) as well as the rest of the compared region-based segmentation approaches (discussed above). Fig 5 shows the output for the detection of epithelial and stromal regions for the same six core tissue images. The first row (from the top) shows the original scanned image, the second row is the gold-standard and the third row shows our segmentation results. The remaining rows depict the results obtained by the various methods discussed above.

As shown, our vcells-MM segmentation provides an efficient initial binary partitioning which removes the background area while maintaining an effective segmentation of the core tissue image. Note that, the watershed yields an excessive number of segments which leads to high spatial complexity in image and therefore poor accuracy (JI). Furthermore, the vcells-MM segmentation produces more natural regions than the ones produced by the superpixels (with more compact and uniform segments). Regarding the detection of epithelial and stromal regions our method avoids the over-segmentation results (occurring in the *watershed*, *pixel-based* methods, *Otsu thresholding* and *no-threshold segmentation*) as well as the under-segmentation results (observed in the *waterfall L2* and the *maximum entropy multi-thresholding*).

## 7 Discussion

The proposed unsupervised framework for segmentation of epithelial and stromal regions in H&E images offers several advantages over existing methods. Unlike the current supervised segmentation approaches, our data-independent segmentation doesn’t require predefined training data of hand-labelled region annotations. However, it could be argued that the average recognition accuracy obtained here (F1 score of 80-81%) is somewhat lower than the average accuracy reported with supervised methods (e.g. [4–7]). This is due to the fact that clustering methods do not take advantage of any predefined manual annotations. So while they can be used to alleviate the burden of the predefined manual annotation, this is often at the cost of slightly inferior accuracy. Segmentation of microscopy images independently (or in absence) of a manually-annotated image is particularly needed in real-time systems where a large variety of images with undefined content and no gold-standard need to be segmented.

Unlike the individual clustering algorithms, the use of CC enabled a more robust detection of tissue regions in our data set. The procedure attempts to emulate real-life strategies which often arise when experts do not agree about a particular problem. In such cases, experts meet to discuss all known aspects of a case in order to reach a consensus. As illustrated in Fig 3, it is difficult to select one best clustering method that is consistently superior across all images and although the *k*-means algorithm showed promising results in most cases, it failed to perform well in the third case from the top. By contrast, although the LVQ method produced poor results in most cases, it provided good results in at least four cases. These results also illustrate the difficulty of standardising clustering algorithms and the advantages of CC in the context of histological image unsupervised analysis.

In contrast to the compared region-based segmentation methods, the proposed segmentation approach helps to decrease the spatial complexity of the image while appearing to retain important information about tissue compartments. This also improves the visualization and hence the interpretation of the image. It is also computationally less expensive when compared to the popular *watershed* method.

Although the histogram-based and the pixel-based segmentation methods do not require prior binary initial segmentations, they neglect the spatial information in the image as they only rely on the intensity of pixels. This fact explains their poor segmentation results attained in our experiments. Furthermore, the histogram-based algorithms are sensitive to noise and often fail in images with no defined distribution peaks.

## 8 Conclusion

We proposed an integrated framework for unsupervised segmentation of epithelial and stromal tissues, in haematoxylin and eosin (H&E) stained sections from tissue micro-array (TMA) cores. This involves an initial segmentation algorithm, based on mathematical morphology techniques, to partition the digitized images into binary virtual-cells. Colour and morphological features were extracted and selected for the discrimination between the two tissue compartments. This is achieved by utilizing a selective Consensus Clustering (CC) technique, which considers the consensual opinion of a group of clustering algorithms to provide a single more reliable and stable clustering result. For this, we exploited two CC functions, the EAC and the voting-based. For the latter, we introduced a label matching technique which imposed consistency to the different base clustering outcomes.

The strength of the proposed framework is derived from the fact that (a) it provides a data annotation-independent recognition model, (b) it facilitates the interpretation and visualization of the image by reducing its spatial complexity, while retaining its essential histopathological contents, (c) unlike other standard segmentation models our framework avoids over-/under-segmentations, (d) the CC algorithm incorporates the capability of multiple clustering models and hence provides more robust unsupervised identification of different tissue compartments, and (e) qualitative and quantitative results tested on a set of forty-five hand-segmented H&E stained tissue images verified that our segmentation method outperforms in accuracy and stability current individual clustering approaches. It also compares favourably with commonly used segmentation methods.

## Supporting information

S1 Table Detailed quantitative results of the individual and the consensus clustering approaches. The table displays the average evaluation measures (across the forty-five tested images) for each run of the individual clustering methods. Performance is evaluated in terms of Rand Index, Precision, Recall, F1-score and Jaccard Index.
(PDF)

S2 Table Results table with the morphology and colour features of the vcells (S10 Fig) as well as the morphology features of the nuclei contained in each vcell (S9 Fig). (XLS)

S1 Fig Example (1): Overview process of the unsupervised segmentation of tissue compartments in an example H&E stained core tissue images. (TIF)

S2 Fig Example (2): Overview process of the unsupervised segmentation of tissue compartments in an example H&E stained core tissue images. (TIF)

S3 Fig Example (3): Overview process of the unsupervised segmentation of tissue compartments in an example H&E stained core tissue images. (TIF)

S4 Fig Example (4): Overview process of the unsupervised segmentation of tissue compartments in an example H&E stained core tissue images. (TIF)

S5 Fig Time performance (in milliseconds) of the EAC and the Voting-based consensus clustering methods across the forty-five tested images. (TIF)

S6 Fig One anonymized example image *I* (in full resolution) of haematoxylin and eosin stained section of a TMA core. (TIF)

S7 Fig The stained RGB image I* of S6 Fig in full resolution. (TIF)

S8 Fig The foreground mask of S6 Fig in full resolution. (TIF)

S9 Fig The nuclear markers (seeds in white) of S6 Fig in full resolution. (TIF)

S10 Fig The segmented image *S* with v-cells-MM of S6 Fig in full resolution. (TIF)

S11 Fig Detection of epithelial and stromal regions in S6 Fig using the EAC method. (TIF)

S12 Fig Detection of epithelial and stromal regions in S6 Fig using the Voting-based method. (TIF)

S13 Fig Hand-segmented gold standard image of S6 Fig in full resolution. (TIF)

S1 Source-code Source code of the proposed methods. A compressed file that contains (a) ImageJ macro for the binary image segmentation algorithm and (b) Java ImageJ plugin for the consensus clustering methods.
(ZIP)

## References

[1] Gurcan MN, Boucheron LE, Can A, Madabhushi A, Rajpoot NM, Yener B. Histopathological image analysis: A review. IEEE Rev Biomed Eng. 2009;2:147–171. doi: 10.1109/RBME.2009.2034865 20671804 10.1109/RBME.2009.2034865 PMC2910932

[2] Madabhushi A, Lee G. Image analysis and machine learning in digital pathology: Challenges and opportunities. Med Image Anal. 2016;33:170–175. doi: 10.1016/j.media.2016.06.037 27423409 10.1016/j.media.2016.06.037 PMC5556681

[3] Rogojanu R, Thalhammer T, Thiem U, Heindl A, Mesteri I, Seewald A, et al Quantitative Image Analysis of Epithelial and Stromal Area in Histological Sections of Colorectal Cancer: An Emerging Diagnostic Tool. BioMed Research International. 2015;2015(569071). doi: 10.1155/2015/569071 26579535 10.1155/2015/569071 PMC4633538

[4] Linder N, Konsti J, Turkki R, Rahtu E, Lundin M, Nordling S, et al Identification of tumor epithelium and stroma in tissue microarrays using texture analysis. Diagn Pathol. 2012;7(1):22 doi: 10.1186/1746-1596-7-22 22385523 10.1186/1746-1596-7-22 PMC3315400

[5] Hiary H, Alomari RS, Saadah M, Chaudhary V. Automated segmentation of stromal tissue in histology images using a voting Bayesian model. Signal Image Video P. 2013;7(6):1229–1237. doi: 10.1007/s11760-012-0393-2

[6] Xu J, Luo X, Wang G, Gilmore H, Madabhushi A. A Deep Convolutional Neural Network for segmenting and classifying epithelial and stromal regions in histopathological images. Neurocomputing. 2016;191:214–223. doi: 10.1016/j.neucom.2016.01.034 28154470 10.1016/j.neucom.2016.01.034 PMC5283391

[7] Wang CW, Fennell D, Paul I, Savage K, Hamilton P. Robust automated tumour segmentation on histological and immunohistochemical tissue images. PLoS One. 2011;6(4) 10.1371/journal.pone.0015818 PMC3046129 21386898

[8] Choromanska A, Monteleoni C. Online Clustering with Experts. In: Proceedings of the 2011 International Conference on On-line Trading of Exploration and Exploitation 2—Volume 26. OTEAE’11. JMLR.org; 2011. p. 1–18.

[9] Datar M, Padfield D, Cline H. Color and texture based segmentation of molecular pathology images usING HSOMS. In: 2008 5th IEEE International Symposium on Biomedical Imaging: From Nano to Macro. IEEE; 2008. p. 292–295.

[10] Chen B, Mete M, Kockara S. Parameter-free multi-level fuzzy c-means clustering for unsupervised structure detection in histological images. In: SDPS 2010 Transformative Systems Conference, Dallas, USA. Citeseer; 2010.

[11] Naqvi S, Garibaldi JM. An Investigation into the use of Fuzzy C-Means Clustering of Fourier Transform Infrared Microscopic Data for the Automation of Breast Cancer Grading. In: Intelligent Modeling and Analysis (IMA) Research Group, School of Computer Science, University of Nottingham. Citeseer; 2009. p. 1–6.

[12] Jain AK, Murty MN, Flynn PJ. Data clustering: a review. ACM Comput Surv. 1999;31(3):264–323. doi: 10.1145/331499.331504

[13] Fraley C, Raftery AE. How many clusters? Which clustering method? Answers via model-based cluster analysis. Comput J. 1998;41(8):578–588. doi: 10.1093/comjnl/41.8.578

[14] Fred AL, Jain AK. Combining multiple clusterings using evidence accumulation. IEEE Trans Pattern Anal Mach Intell. 2005;27(6):835–850. doi: 10.1109/TPAMI.2005.113 15943417 10.1109/TPAMI.2005.113

[15] Vega-Pons S, Ruiz-Shulcloper J. A survey of clustering ensemble algorithms. Int J Pattern Recogn. 2011;25(03):337–372. doi: 10.1142/S0218001411008683

[16] Topchy AP, Law MHC, Jain AK, Fred AL. Analysis of consensus partition in cluster ensemble. In: ICDM’04. Fourth IEEE International Conference on Data Mining; 2004. p. 225–232.

[17] Khan AM, El-Daly H, Rajpoot N. RanPEC: Random projections with ensemble clustering for segmentation of tumor areas in breast histology images In: Medical Image Understanding and Analysis (MIUA). Swansea, UK: British Machine Vision Association (BMVA); 2012 p. 17–23.

[18] Cooper LA, Kong J, Gutman DA, Wang F, Gao J, Appin C, et al Integrated morphologic analysis for the identification and characterization of disease subtypes. J Am Med Inform Assoc. 2012;19(2):317–323. doi: 10.1136/amiajnl-2011-000700 22278382 10.1136/amiajnl-2011-000700 PMC3277636

[19] Soille P. Morphological image analysis: principles and applications. Springer Science & Business Media; 2013.

[20] Fouad S, Randell D, Galton A, Mehanna H, Landini G, Unsupervised Superpixel-based Segmentation of Histopathological Images with Consensus Clustering In: Medical Image Understanding and Analysis (MIUA). Edinburgh, UK: Springer Volume 723 of the Communications in Computer and Information Science series; 2017, p. 767–779.

[21] Landini G, Randell D, Fouad S, Galton A. Automatic thresholding from the gradients of region boundaries. J Microsc. 2016;00:1–13. 10.1111/jmi.12474 PMC6850295 27649382

[22] Gunduz-Demir C, Kandemir M, Tosun AB, Sokmensuer C. Automatic segmentation of colon glands using object-graphs. Med Image Anal. 2010;14(1):1–12. doi: 10.1016/j.media.2009.09.001 19819181 10.1016/j.media.2009.09.001

[23] Shih FY, Cheng S. Automatic seeded region growing for color image segmentation. Image Vision Comput. 2005;23(10):877–886. doi: 10.1016/j.imavis.2005.05.015

[24] Zhou X, Li F, Yan J, Wong ST. A novel cell segmentation method and cell phase identification using Markov model. IEEE T Inf Technol B. 2009;13(2):152–157. doi: 10.1109/TITB.2008.2007098 10.1109/TITB.2008.2007098 PMC2846548 19272857

[25] Wang Y, Seguro F, Kao E, Zhang Y, Faraji F, Zhu C, Haraldsson H, Hope M, Saloner D and Liu J Segmentation of lumen and outer wall of abdominal aortic aneurysms from 3D black-blood MRI with a registration based geodesic active contour model. Med Image Anal. 2017;40(1):1–10. 28549310 10.1016/j.media.2017.05.005 PMC5796767

[26] Fouad S, Landini G, Randell D, Galton A. Morphological Separation of Clustered Nuclei in Histological Images. In: Campilho A, Karray F, editors. Image Analysis and Recognition: 13th International Conference, ICIAR 2016, Póvoa de Varzim, Portugal, July 13-15, 2016, Proceedings. Springer International Publishing; 2016. p. 599–607.

[27] Hartigan JA, Wong MA. Algorithm AS 136: A k-means clustering algorithm. J R Stat Soc Ser C Appl Stat. 1979;28(1):100–108.

[28] Ruifrok AC, Johnston DA, et al Quantification of histochemical staining by color deconvolution. Anal Quant Cytol Histol. 2001;23(4):291–299. 11531144

[29] Lambert JH. Photometria, sive de Mensura et gradibus luminis, colorum et umbrae (Augsberg: Eberhard Klett). Sumptibus viduae Eberhardi Klett, typis Christophori Petri Detleffsen; 1760.

[30] Huang LK, Wang MJJ. Image thresholding by minimizing the measures of fuzziness. Pattern Recogn. 1995;28(1):41–51. doi: 10.1016/0031-3203(94)E0043-K

[31] Beucher S, Lantuéjoul C. Use of Watersheds in Contour Detection; 1979. International Workshop on Image Processing: Real-time Edge and Motion Detection/Estimation, Rennes, France.

[32] Achanta R, Smith K, Lucchi A, Fua P, Süsstrunk S. SLIC Superpixels; 2010. Technical report, EPFL no. 149300.

[33] Achanta R, Shaji A, Smith K, Lucchi A, Fua P, Susstrunk S. SLIC Superpixels Compared to State-of-the-Art Superpixel Methods. IEEE Trans Pattern Anal Mach Intell. 2012;34(11):2274–2282. doi: 10.1109/TPAMI.2012.120 22641706 10.1109/TPAMI.2012.120

[34] Vincent L. Morphological grayscale reconstruction in image analysis: applications and efficient algorithms. IEEE Trans Image Process. 1993;2(2):176–201. doi: 10.1109/83.217222 18296207 10.1109/83.217222

[35] Landini G, Othman IE. Architectural analysis of oral cancer, dysplastic, and normal epithelia. Cytometry A. 2004;61(1):45–55. doi: 10.1002/cyto.a.20082 15351988 10.1002/cyto.a.20082

[36] Landini G, Randell DA, Breckon TP, Han JW. Morphologic characterization of cell neighborhoods in neoplastic and preneoplastic epithelium. Anal Quant Cytol Histol. 2010;32(1):30–38. 20701085

[37] Landini G. Advanced shape analysis with ImageJ. Proceedings of the Second ImageJ User and Developer Conference, Luxembourg, 6-7 November, 2008. p116-121. Plugins available from: http://www.mecourse.com/landinig/software/software.html.

[38] Hall MA. Correlation-based Feature Subset Selection for Machine Learning. University of Waikato Hamilton, New Zealand; 1998.

[39] Kohonen T. In: Learning Vector Quantization. Berlin, Heidelberg: Springer Berlin Heidelberg; 1995 p. 175–189.

[40] Dempster AP, Laird NM, Rubin DB. Maximum likelihood from incomplete data via the EM algorithm. J R Stat Soc Ser B Stat Methodol. 1977; p. 1–38.

[41] Ester M, Kriegel HP, Sander J, Xu X, et al. A density-based algorithm for discovering clusters in large spatial databases with noise. In: Int’l Conference on Knowledge Discovery in Databases and Data Mining (KDD-96), Portland, Oregon. vol. 96. AAAI Press, Menlo Park, CA (United States); 1996. p. 226–231.

[42] Defays D. An efficient algorithm for a complete link method. Comput. J. Br Computer Soc. 1977;20(4):364–366.

[43] Fern XZ, Lin W. Cluster ensemble selection. Stat Anal Data Min. 2008;1(3):128–141. doi: 10.1002/sam.10008

[44] Hadjitodorov ST, Kuncheva LI, Todorova LP. Moderate diversity for better cluster ensembles. Inform Fusion. 2006;7(3):264–275. doi: 10.1016/j.inffus.2005.01.008

[45] Hubert L, Arabie P. Comparing partitions. J Classif. 1985;2(1):193–218. doi: 10.1007/BF01908075

[46] Rasband WS. ImageJ. US National Institutes of Health, Bethesda, Maryland, USA (http://imagej.nih.gov/ij/). 1997-2017.

[47] Frank E, Hall M, Witten IH. The WEKA Workbench. Online Appendix for D̈ata Mining: Practical Machine Learning Tools and Techniques 4th ed. Morgan Kaufmann; 2016.

[48] Powers D M W. Evaluation: From Precision, Recall and F-Measure to ROC, Informedness, Markedness & Correlation. Journal of Machine Learning Technologies. 2011;2(1):37–63.

[49] Beucher S. Watershed, Hierarchical Segmentation and Waterfall Algorithm In: Serra J, Soille P, editors. Mathematical Morphology and Its Applications to Image Processing. Dordrecht: Springer Netherlands; 1994 p. 69–76.

[50] Sage D. An ImageJ plugin to apply watershed segmentation on graylevel images; 2011. Available from: http://bigwww.epfl.ch/sage/soft/watershed/.

[51] Borovec KJ J. jSLIC: superpixels in ImageJ; 2014. Computer Vision Winter Workshop. Praha.

[52] Sacha J. IJ Plugins: k-means Clustering;. Available from: http://ij-plugins.sourceforge.net/plugins/segmentation/k-means.html.

[53] Liao P-S, Chung P-C. A fast algorithm for multilevel thresholding. J Inf Sci Eng. 2001;17(5):713–727

[54] Otsu N. A threshold selection method from gray-level histogram. IEEE Transactions on System Man Cybernetics. 1979;9(1):62–66 doi: 10.1109/TSMC.1979.4310076

[55] Tosa Y. IJ Plugins: Multi Otsu Threshold. Plugins available from: http://imagej.net/Multi_Otsu_Threshold.

[56] Bonnet N, Cutrona J, Herbin M. A ‘no-threshold’ histogram-based image segmentation method. Pattern Recognition. 2002; vol. 35:2319–2322 doi: 10.1016/S0031-3203(02)00057-2

[57] Kapur J N, Sahoo P K, Wong A K C. IJ Plugins: Segmentation fuzzy 514 V2 Plugins available from: https://imagej.nih.gov/ij/plugins/inserm514/Documentation/Segmentation_fuzzy_514/Segmentation_fuzzy_514.html

[58] Kapur J N, Sahoo P K, Wong A K C. A new method for gray-level picture thresholding using the entropy of the histogram. Computer Vision, Graphics, and Image Processing. 2002;29(3):273–285 doi: 10.1016/0734-189X(85)90125-2

