# Supplementary material for: Unsupervised morphological segmentation of tissue compartments in histopathological images
Source: PLoS One. 2017 Nov 30;12(11):e0188717. doi: 10.1371/journal.pone.0188717 (PMC5708642; doi:10.1371/journal.pone.0188717)
Supplement: S1 Table — The table displays the average evaluation measures (across the forty-five tested images) for each run of the individual clustering methods. Performance is evaluated in terms of Rand Index, Precision, Recall, F1-score and Jaccard Index. (PDF) [file pone.0188717.s001.pdf]

Performance evaluation of the consensus clustering methods (EAC and Voting-based displayed in the last two rows) compared against five individual clustering approaches

For every individual clustering algorithm and for each run we evaluated the evaluation measures across the forty-five test images then we estimated the average result across its runs

All algorithms initial segmentations were performed via our vcells-MM method.

Performance was evaluated in terms of Rand Index, Precision, Recall, F1-score and Jaccard Index

Clustering Algorithm	True Positive	True Negative	False Positive	False Negative	Rand Index (RI)	Precision (P)	Recall (R)	F1 score (F1)	Jaccard Index (JI)
Kmeans_seed_value 1	1268144.20454545	643113.204545455	422188.272727273	197668.113636364	0.7536863636	0.7523886364	0.8301818182	0.7868840909	0.7061284091
Kmeans_seed_value 2	1264937.84090909	633856.545454545	425394.636363636	206924.772727273	0.7467840909	0.7485931818	0.8231181818	0.7813568182	0.6961113636
Kmeans_seed_value 3	1206568.95454545	628981.454545455	483763.522727273	211799.863636364	0.7380159091	0.7395659091	0.8226295455	0.7754431818	0.6836420455
Kmeans_seed_value 4	1244795.22727273	620674.090909091	445537.25	220107.227272727	0.7432727273	0.7436522727	0.8203113636	0.7774840909	0.6933375
Kmeans_seed_value 5	1206091.38636364	629199.840909091	484241.090909091	211581.477272727	0.7385409091	0.7400181818	0.8233954545	0.7759931818	0.6843511364
Kmeans_seed_value 6	1197984.45454545	617896.545454545	492348.022727273	222884.772727273	0.7314068182	0.7413522727	0.8163545455	0.7721204545	0.6737852273
Kmeans_seed_value 7	1276118.27272727	642958.25	414214.204545455	197823.068181818	0.7545954545	0.7542022727	0.8304113636	0.7756595	0.5820852273
Kmeans_seed_value 8	1266290.90909091	634689.022727273	424041.568181818	206092.295454545	0.7477363636	0.7493636364	0.8239522727	0.772175	0.6972784091
Kmeans_seed_value 9	1195197.97727273	627151.477272727	495134.5	213629.840909091	0.7371295455	0.7390454545	0.8221227273	0.7748227273	0.6831113636
Kmeans_seed_value 10	1195071.25	626772.159090909	495261.227272727	214009.159090909	0.7363090909	0.7380909091	0.8212977273	0.7739727273	0.6818670455
**Average_of_Kmeans (over all runs)**	**1232120.04772727**	**630529.259090909**	**458212.429545455**	**210252.059090909**	**0.7427477273**	**0.7446272727**	**0.8233775**	**0.7765911773**	**0.6781697727**
EM seed_value 1	1205038.04545455	610707.022727273	485294.431818182	230074.295454545	0.7321818182	0.7275568182	0.8212159091	0.7685863636	0.670925
EM seed_value 2	1212620.86363636	614635.454545455	477711.613636364	226145.863636364	0.7393863636	0.7357454545	0.8242227273	0.7747340909	0.6770477273
EM seed_value 3	1204649.34090909	610441.181818182	485683.136363636	230340.136363636	0.7349272727	0.7298840909	0.8228181818	0.7707431818	0.6741659091
EM seed_value 4	1214892.27272727	610074.318181818	475440.204545455	230707	0.7370818182	0.7335431818	0.8223613636	0.7727090909	0.6751386364
EM seed_value 5	1221387	620654.386363636	468945.477272727	220126.931818182	0.7374659091	0.7328295455	0.8242454545	0.7730477273	0.6767818182
EM seed_value 6	1198642.95454545	604319.409090909	491689.522727273	236461.909090909	0.7342931818	0.73075	0.8210704545	0.7704454545	0.6740227273
EM seed_value 7	1187119.77272727	603188.340909091	503212.704545455	237592.977272727	0.7311704545	0.7270295455	0.8202295455	0.7677295455	0.6692011364
EM seed_value 8	1204755.65909091	610359.409090909	485576.818181818	230421.909090909	0.7335795455	0.7287909091	0.8211659091	0.7694045455	0.6717022727
EM seed_value 9	1208148.18181818	611932.545454545	482184.295454545	228848.772727273	0.736925	0.7322522727	0.82275	0.7722136364	0.6755670455
EM seed_value 10	1201984.47727273	608688.136363636	488348	232093.181818182	0.7360454545	0.7315909091	0.8230568182	0.7718681818	0.6738034091
**Average_of_EM (over all runs)**	**1205923.85681818**	**610500.020454545**	**484408.620454546**	**230281.297727273**	**0.7353056818**	**0.7309972727**	**0.8223136364**	**0.7711481818**	**0.6738355682**
AH link type and distance 1	1341612.31818182	520385.818181818	348720.159090909	320395.5	0.7327386364	0.7881863636	0.7772977273	0.7688136364	0.6317681818
AH link type and distance 2	1341612.31453455	520385.818181818	348720.159090909	320395.5	0.7327386364	0.7881863636	0.7772977273	0.7688136364	0.6317681818
AH link type and distance 3	1241612.31818182	520385.818181818	348720.159090909	320395.5	0.7327386364	0.7881863636	0.7772977273	0.7688136364	0.6317681818
AH link type and distance 4	1341612.31818182	520385.818181818	348720.159090909	320395.5	0.7327386364	0.7881863636	0.7772977273	0.7688136364	0.6317681818
AH link type and distance 5	1341612.31818182	520385.818181818	348720.159090909	320395.5	0.7327386364	0.7881863636	0.7772977273	0.7688136364	0.6317681818
AH link type and distance 6	1341612.31818182	520385.818181818	348720.159090909	320395.5	0.7327386364	0.7881863636	0.7772977273	0.7688136364	0.6317681818
**Average_of_AH (over all runs)**	**1324945.65090728**	**520385.818181818**	**348720.159090909**	**320395.5**	**0.7327386364**	**0.7881863636**	**0.7772977273**	**0.7688136364**	**0.6317681818**
LVQ learning rate 1	1602718.88636364	114449.613636364	87613.5909090909	726331.704545455	0.6578909091	0.9310022727	0.6710909091	0.7673590909	0.4925909091
LVQ learning rate 2	1609020.06818182	107860.590909091	81312.4090909091	732920.727272727	0.6566545455	0.9036181818	0.6688909091	0.7672386364	0.3752168182
LVQ learning rate 3	1608685.65909091	106698.090909091	81646.8181818182	734083.227272727	0.6548863636	0.912465464	0.6670318182	0.7657954545	0.3749418182
LVQ learning rate 4	1609391.09090909	112984.977272727	80941.3863636364	727796.340909091	0.659475	0.9125522727	0.67155	0.7684863636	0.3813418182
**Average_of_LVQ (over all runs)**	**1607453.92613637**	**110498.318181818**	**82878.5511363637**	**730283**	**0.6572267045**	**0.9149095478**	**0.6696409091**	**0.7672198864**	**0.4060228409**
**MDB**	**1168147.09090909**	**633504.954545455**	**423185.386363636**	**207276.363636364**	**0.7407762124**	**0.7340685291**	**0.8492999643**	**0.7874911976**	**0.7043909091**
**Consensus Clustering EAC**	**1304569.45454545**	**651994.227272727**	**385763.022727273**	**188787.090909091**	**0.7730050246**	**0.771782754**	**0.8463582038**	**0.8073519952**	**0.7343045455**
**Consensus Clustering Voting-based**	**1313915.86363636**	**652302.636363636**	**385416.613636364**	**188478.681818182**	**0.7740670924**	**0.7831952877**	**0.8454781276**	**0.8131458145**	**0.7419318182**
