# Supplementary figures and images for: Unsupervised morphological segmentation of tissue compartments in histopathological images
Source: PLoS One. 2017 Nov 30;12(11):e0188717. doi: 10.1371/journal.pone.0188717 (PMC5708642; doi:10.1371/journal.pone.0188717)

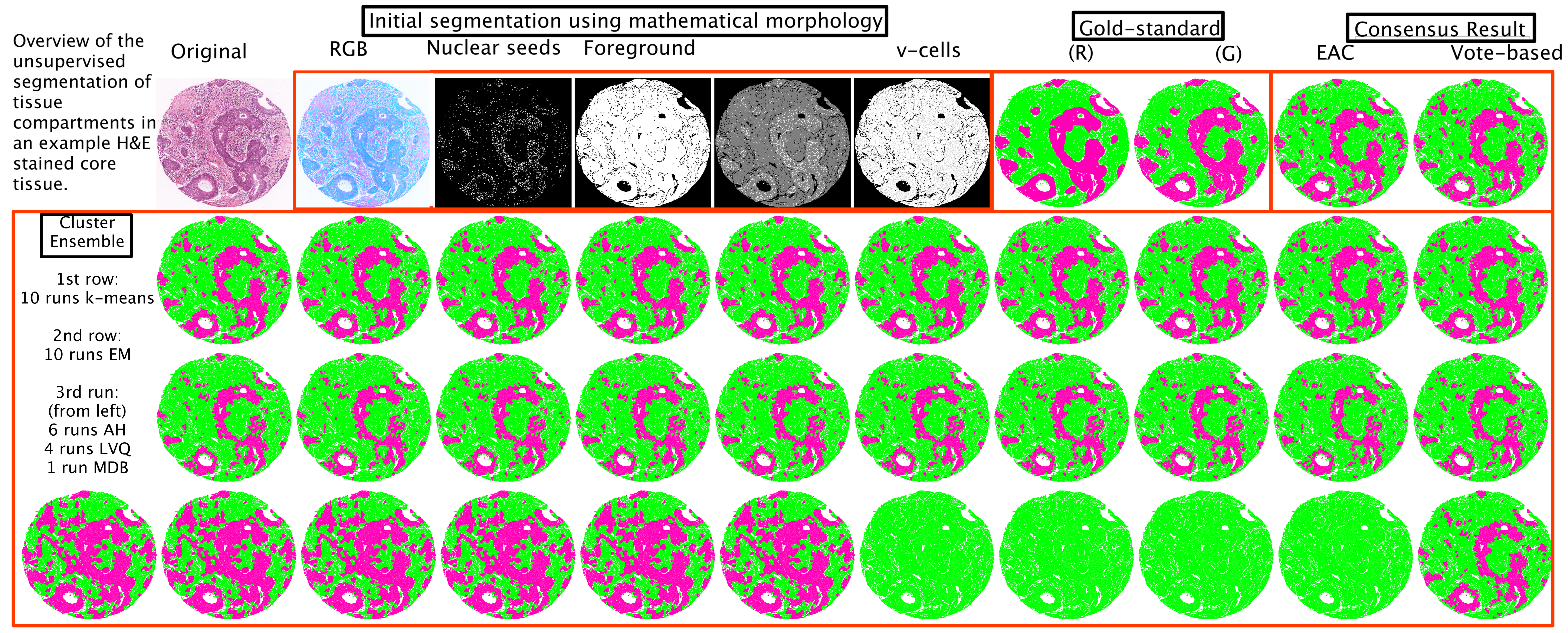

Supplement: S3 Fig — (TIF) [file pone.0188717.s005.tif]

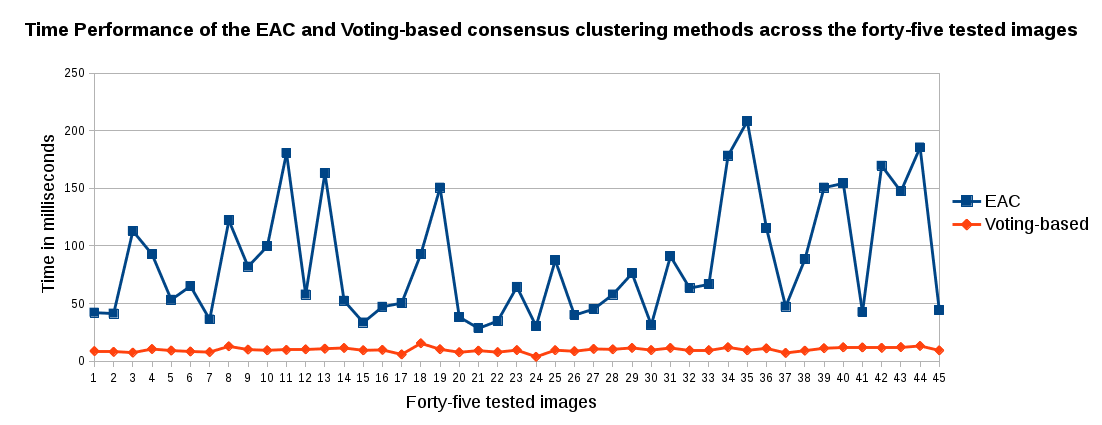

Supplement: S5 Fig — (TIF) [file pone.0188717.s007.tif]

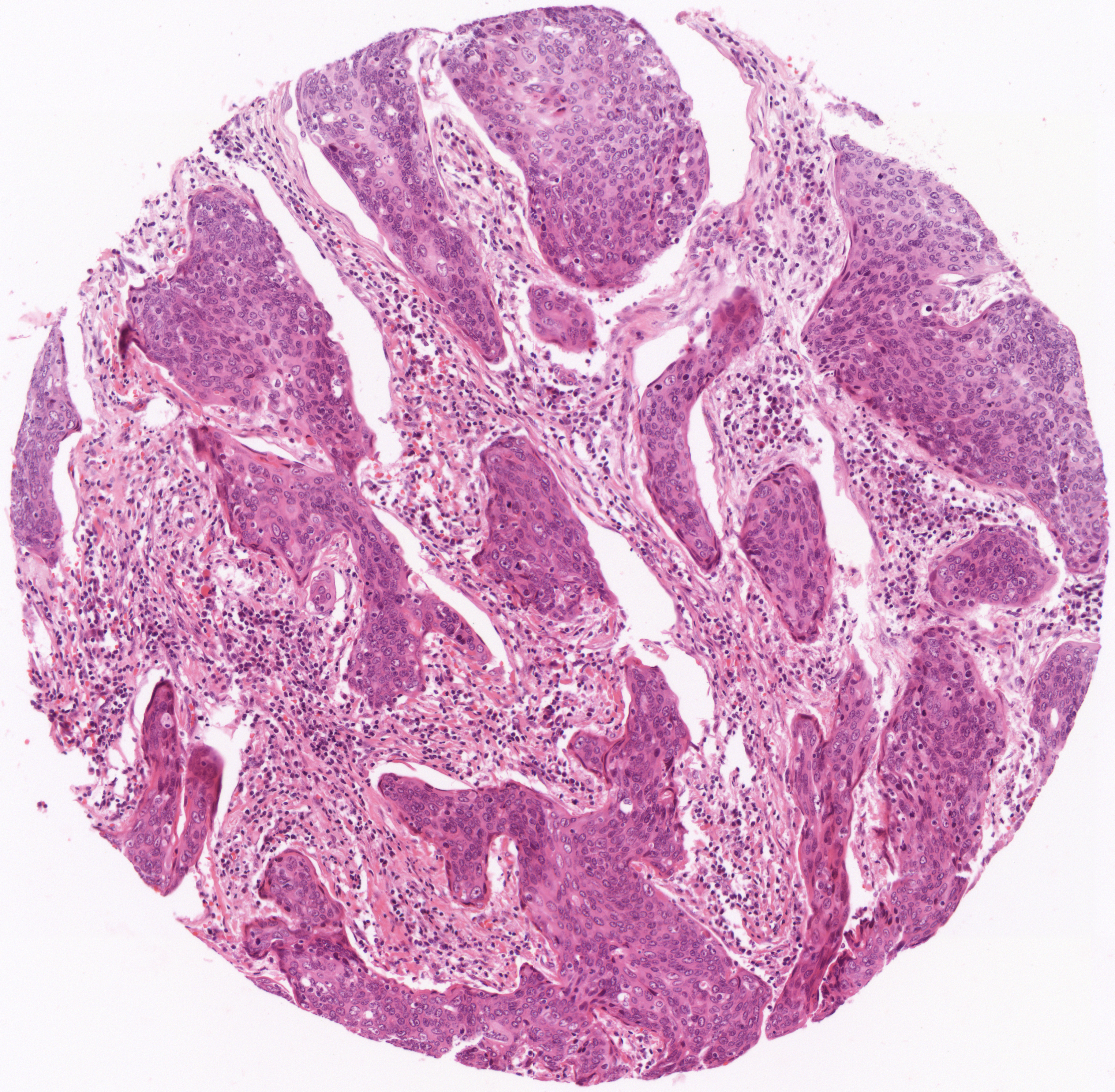

Supplement: S6 Fig — (TIF) [file pone.0188717.s008.tif]

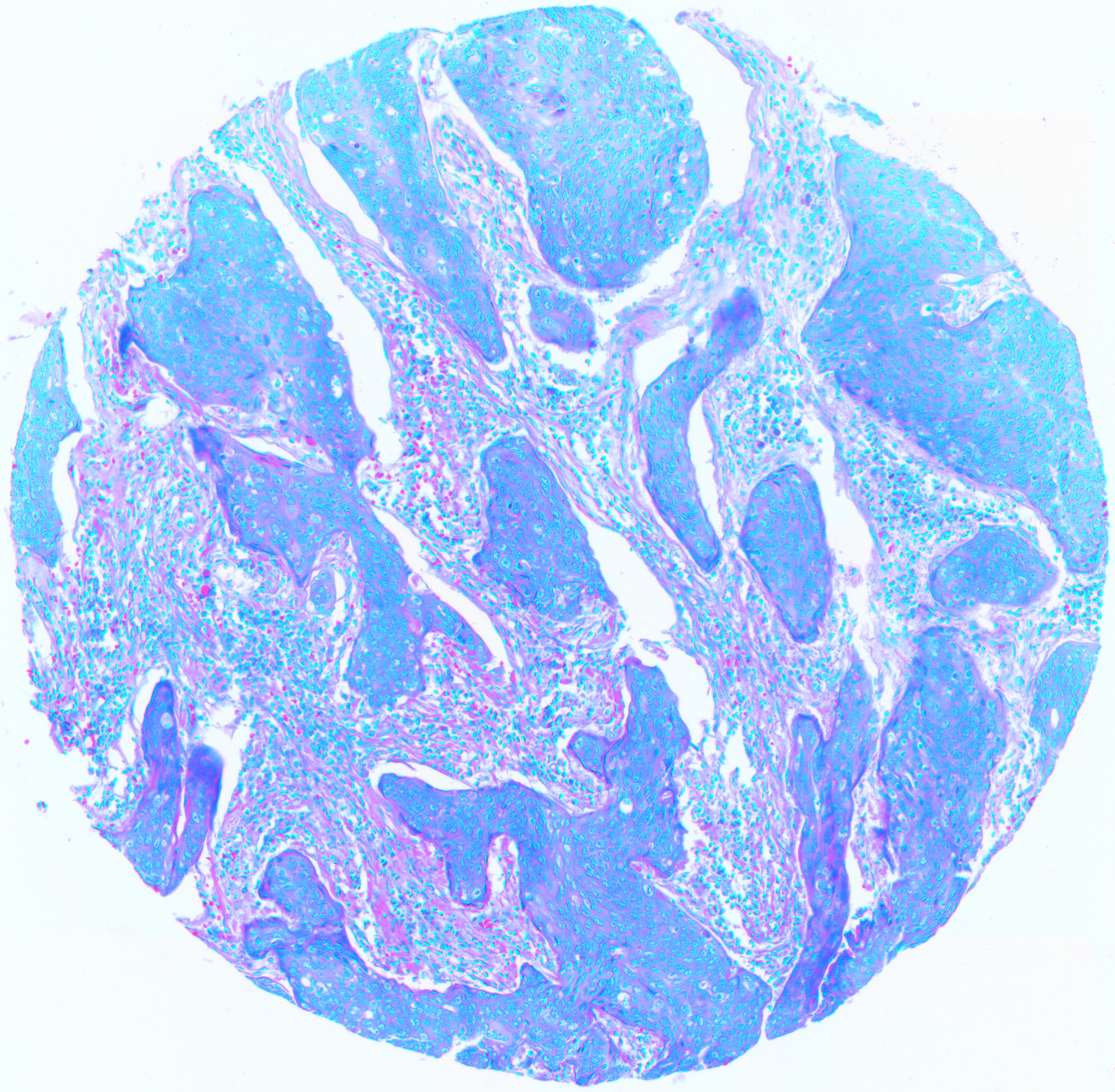

Supplement: S7 Fig — (TIF) [file pone.0188717.s009.tif]

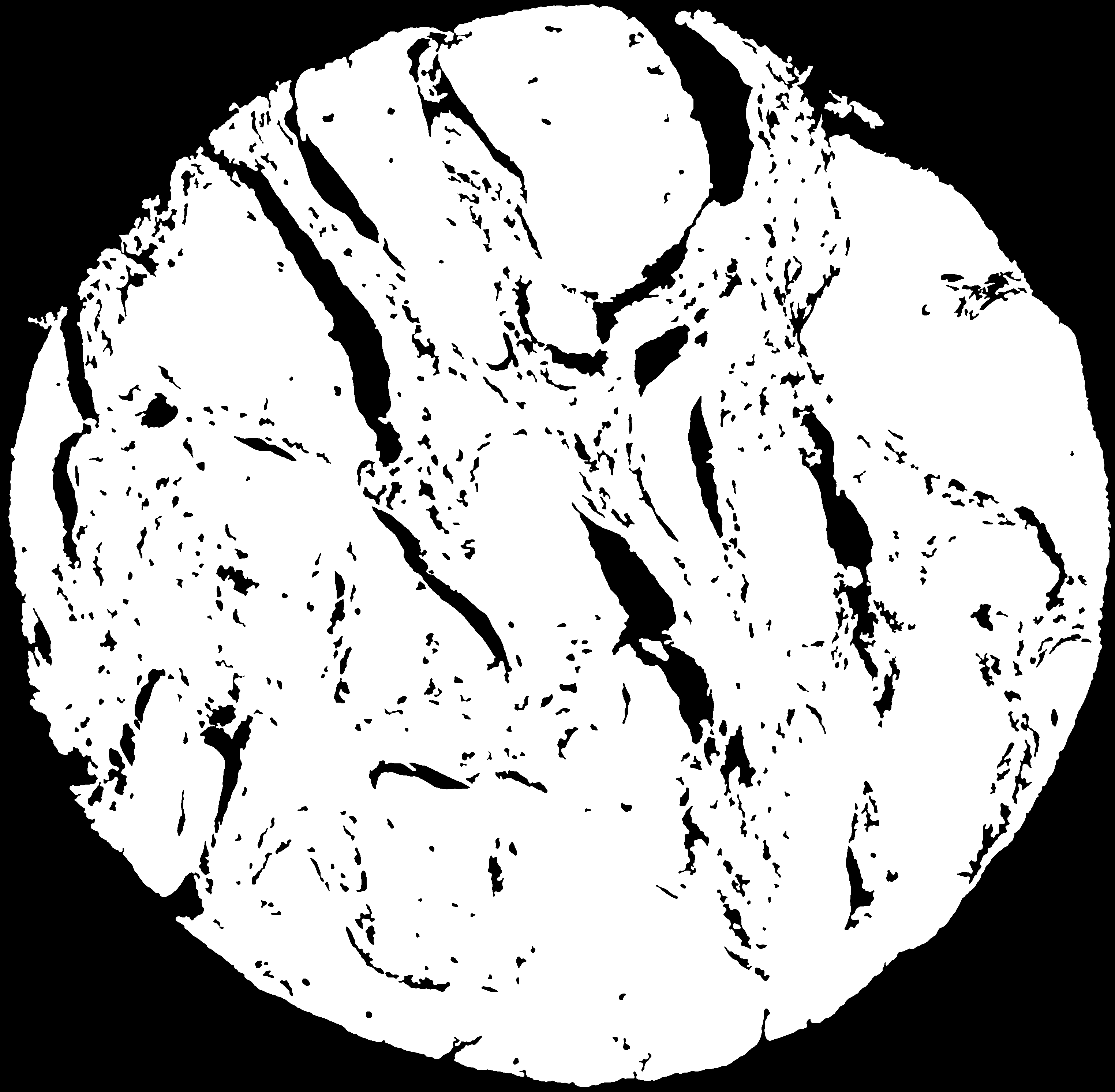

Supplement: S8 Fig — (TIF) [file pone.0188717.s010.tif]

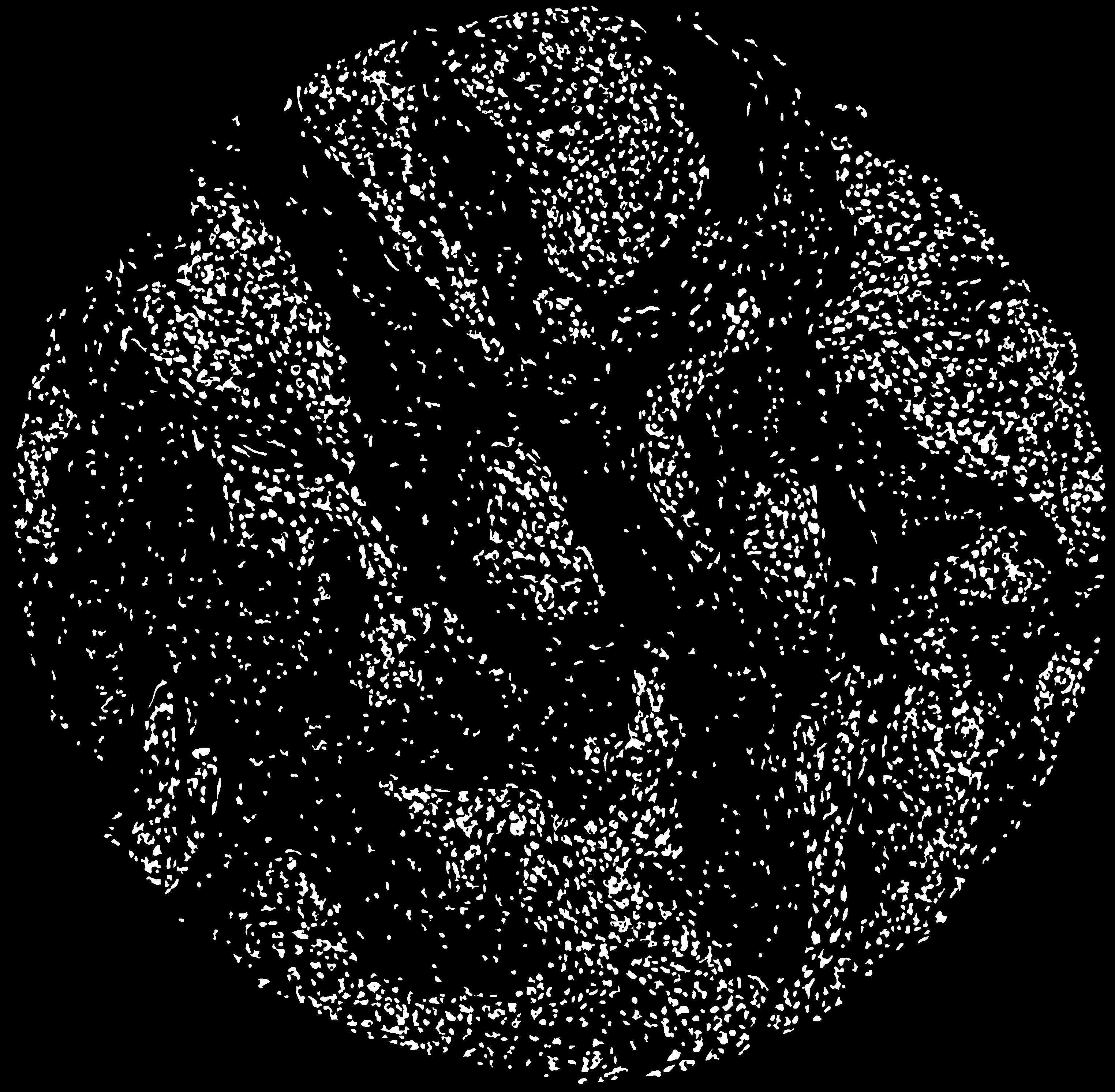

Supplement: S9 Fig — (TIF) [file pone.0188717.s011.tif]

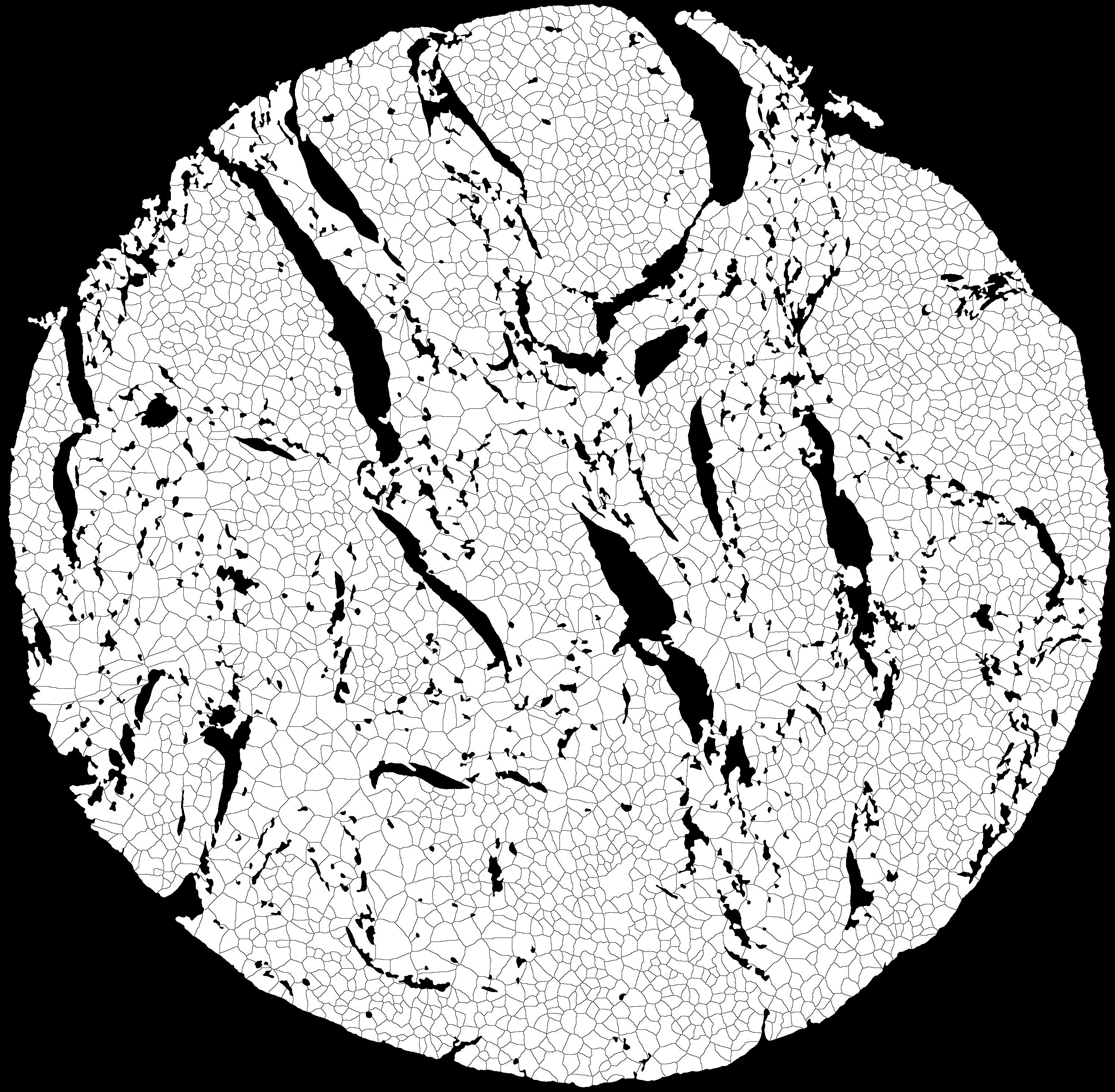

Supplement: S10 Fig — (TIF) [file pone.0188717.s012.tif]
